# Development of a preliminary multivariable model predicting hamstring strain injuries during preseason screening in soccer players: a multidisciplinary approach

**DOI:** 10.1080/07853890.2025.2494683

**Published:** 2025-05-08

**Authors:** Diane Baize, Stéphanie Mériaux-Scoffier, Anasthase Massamba, Thomas Hureau, Nicolas Reneaud, Yoann Garcia—Gimenez, Florian Marchand, Bastien Bontemps, Baptiste Corcelle, Vincent Maléjac, Amyn Jaafar, Emiliano Ippoliti, Florian Payet, Iliès Ajarai, Fabienne d’Arripe-Longueville, Enzo Piponnier

**Affiliations:** ^a^ LAMHESS UPR 6312, Université Côte d’Azur, Nice, France; ^b^ CEERIPE UR 3072, Université de Strasbourg, Strasbourg, France; ^c^ Racing Club de Strasbourg Alsace, Strasbourg, France; ^d^ CHU de Nice, Université Côte d’Azur, Nice, France; ^e^ CHU de Nîmes, Nîmes, France; ^f^ LIBM EA 7424, Université Savoie Mont Blanc, Chambéry, France; ^g^ LIBM EA 7424, Université Jean Monnet, Saint Priest en Jarez, France; ^h^ Racing Club Pays de Grasse, Grasse, France; ^i^ AS Cagnes-Le Cros Football, Cagnes-sur-mer, France; ^j^ Olympique Gymnaste Club of Nice Côte d’Azur, Nice, France; ^k^ Olympique Lyonnais, Décines-Charpieu, France

**Keywords:** European football, performance fatigability, primary prevention, psychological factors, sports injuries

## Abstract

**Objective:**

Reducing the incidence of hamstring strain injuries (HSIs) is a priority for soccer clubs. However, robust multifactorial predictive models are lacking and potential predictors such as sprint kinematics, performance fatigability, and psychological variables have been overlooked. Thus, the aim of this study was to develop a preliminary parsimonious multifactorial model to predict players at risk of HSI through preseason screening.

**Materials and Method:**

Psychological, physiological, kinematic, performance fatigability and health-related variables were collected for 120 regional and national soccer players during the 2022 preseason. HSIs were prospectively recorded over the entire soccer season. After variable selection, logistic regressions with the Wald backward stepwise method were used to refine the model. The predictive abilities of the model and of the individual variables were determined using the area under the receiver operating characteristic curve (AUC).

**Results:**

Twenty-nine players sustained an HSI during the follow-up period. The final model included eight variables: age, sex, HSI history, knee flexor performance fatigability, sprint performance (best sprint time and maximal theoretical velocity V_0_), perceived vulnerability to injury, and subjective norms in soccer. While its model was preliminary, it showed good fit indices and strong predictive performance (true positive rate: 79%, AUC = .82). None of the variables evaluated independently demonstrated satisfactory performance in predicting HSI (AUC≤.65).

**Conclusion:**

Using a multidisciplinary approach and measurements of only a few variables during preseason screening, the current model tends to demonstrate high accuracy in identifying soccer players at risk of HSI.

## KEY MESSAGES

- The study introduces a preliminary multifactorial model derived from preseason screening, effectively predicting 79% of subsequent HSIs.
- The performance fatigability of the knee flexors, perceived injury vulnerability and internalization of subjective norms about fatigue and pain in soccer play crucial roles in predicting HSI occurrence, alongside other predictors such as age, HSI history and sprint performance.
- Individual HSI predictors, assessed independently, show limited predictive power.

## Introduction

Hamstring strain injuries (HSIs) are among the most frequent and burdensome injuries in amateur and professional soccer [1,2]. HSIs predominantly occur during sprinting [3], specifically in the late-swing phase of the running cycle [4]. Their high occurrence towards the end of each half during matches, suggesting fatigue may influence HSI incidence [5]. HSIs may impact not only the health of the players, but also their careers and the team’s performance [6]. Therefore, from a preventive perspective, numerous risk factors have been investigated independently [7,8]. These studies have explored extrinsic risk factors such as variations in the training load or in sprint exposures, and intrinsic risk factors such as personal characteristics, physiological or neuromuscular factors, and kinetic or kinematic variables. Personal characteristic risk factors usually included age, sex, injury history or playing position. These non-modifiable factors are robust predictors of HSIs [7,8]. Among the modifiable risk factors, physiological and neuromuscular factors are the most considered. These factors include hamstring eccentric force, leg muscle force imbalance, quadriceps to hamstring ratio, knee flexors endurance, range of motion of the lower legs, fascicle length of biceps femoris, intermuscular coordination and core stability [7,8]. Kinetic and kinematic factors have also been investigated. These factors encompass parameters related to the force-velocity profile (FV-profile) such as the maximal theoretical horizontal force F_0_ [9], but also the anterior pelvic tilt and thoracic side bending while sprinting [10,11], and alterations of these parameters during repeated sprints [12]. More recently, researchers also focused on psychological risk factors [13,14]. Athletic identity, competitive motivational goals, persistence through pain, inappropriate recovery strategies and the willingness to exceed the body’s limits were documented as intrinsic psychological risk factors for HSIs.

While the identification of modifiable risk factors constitutes useful knowledge for enhancing prevention strategies on the field, their predictive effectiveness is debated in the literature [7,8,15]. This observation is likely attributed to the isolated or linear measurement approach applied to a multifactorial phenomenon [7,8,16], overlooking the complex interactions between different predictors. For example, a high anxiety level can trigger sleep disturbances, alter the recovery process and resulting in fatigue [17]. Fatigue, in turn, negatively impacts muscle force production, attention and coordination [18], increasing the injury risk during play [19].

Thus, recent research has attempted to consider the different risk factors jointly, determining the predictive power of multifactorial models in identifying players at risk of HSI before it occurs. Using a one-time preseason evaluation, Ruddy et al. [20] considered HSI history, demographic, and eccentric hamstring strength of elite Australian soccer players over 2 seasons. Irrespective of the supervised learning techniques employed to construct the models, these approaches yielded weak predictive power concerning HSI occurrence, likely attributable to the limited scope of the risk factors considered. Ayala et al. [21] using alternating decision tree learning algorithms (ADtrees), developed an efficient model with 96 professional male Spanish soccer players, based on 66 predictors and 10 classifiers (Area Under the Curve (AUC)  =  .837). This model considered personal risk factors, 2 psycho-behavioural risk factors related to fatigue (i.e. sleep quality and burnout), and numerous neuromuscular risk factors (i.e. dynamic postural control, isometric hip abduction and adduction strength, joints ranges of motion, core stability, and isokinetic hamstring and quadricep force) as predictors.

Although effective, this model is challenging to implement in the field and lacked broad applicability to a more diverse range of soccer players. It was based on a specific population of professional male soccer players, required many variables and involved many classifiers. Additionally, some crucial variables were not directly measured (e.g. performance fatigability and sprint related factors). Some well-documented psychological and health-related risk factors for sport injuries were also not tested as predictors (e.g. anxiety traits, avoiding-performance goals, poor coping strategies, neuroticism, perfectionistic strivings, strong athletic identity, persistence through pain, perceived vulnerability, and inappropriate food intake) [19,22–24]. Therefore, one might contemplate whether incorporating a more concise yet pertinent selection of predictors from a broader spectrum of categories into a predictive model could enhance the preseason identification of soccer players vulnerable to HSI. Ideally, this approach should be adaptable for both professional and non-professional players, providing superior predictive accuracy compared to evaluating a single HSI risk factor. The aim of this study was thus to develop a preliminary parsimonious multifactorial model to predict players at risk of HSI through preseason screening. We also evaluated its potential to surpass the accuracy of individual predictor assessments in identifying players prone to HSI. We hypothesized that by incorporating a limited number of diverse factors (i.e. psychological, physiological, kinematic, sprint related and health related characteristics) into the model, we can develop a preliminary effective and parsimonious multifactorial model for predicting soccer players at risk of HSI during preseason screening. This model is expected to achieve predictive performance comparable to more complex multifactorial models previously developed for identifying players prone to HSI [21].

## Material and methods

### Study design

We conducted a prospective cohort study over a complete soccer season. The screening tests were all performed during the preseason (i.e. between July 26 and September 9, 2022). Injury data were then collected until the end of the season (i.e. June, 2023). No experimental intervention was performed following the preseason screening.

### Ethical approval

The protocol was reviewed and granted approval for implementation from the French Committee of Protection of Persons Southwest and overseas III (ID-RCB number: 2021-A02748-33). Measurements were performed in accordance with the ethical standards of the Helsinki Declaration. All participants received an information letter and the consent form at least 1 week before the beginning of the tests. Prior to measurements, signed written informed consent was collected, including parental approval for players under 18.

### Participants

To meet the criteria of a shrinkage factor ≥.8 [25,26] and considering an expected final model integrating 6 to 8 variables (to be easily applicable on the field), the minimal required sample size was estimated to be 134–179 participants. To ensure the estimate of overall risk (model intercept) [25], and based on the epidemiological data of previous studies reporting 13–18% of HSI in their sample [27,28], the minimal sample size was calculated to be 174–227 players. Thus, we planned to include 180 players to build our logistic regression model. The participants were recruited in collaboration with 5 different French soccer clubs including 3 elite soccer club academies, 1 national club, and 1 regional club. Goalkeepers and players presenting psychological disorders, depression according to the Patient Health Questionnaire (PHQ-2), a current injury, or hamstring pain were not included.

### Testing procedure

**Figure 1.**
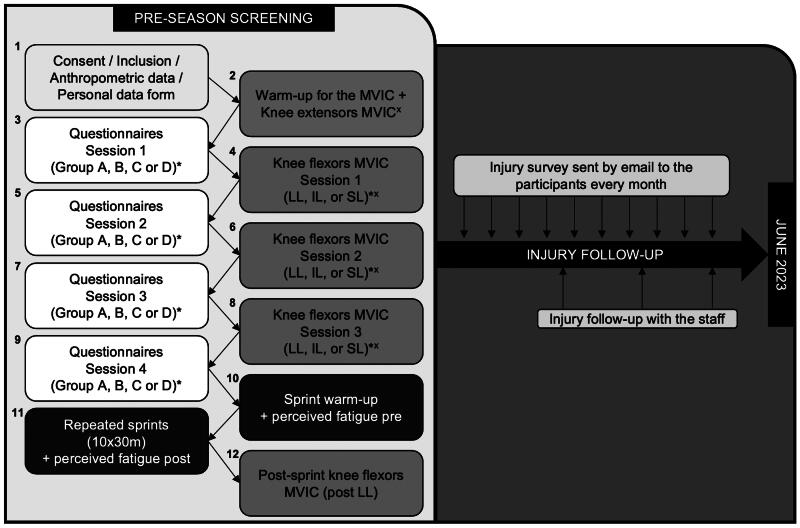
Testing procedure. * = randomized order; ^x^ = randomized order of the first lower limb evaluated. Abbreviations: IL = intermediate length position; LL = long length position; MVIC = maximal voluntary isometric contraction; pre = pre-sprints; post = post-sprints; SL = short length position.

Preseason screening tests contained twelve steps (Figure 1), grouped into 3 parts (questionnaires, maximal voluntary isometric contractions of the lower limbs and repeated sprints). Six experimenters conducted the measurements, with 3 researchers present for all parts leading the testing procedure throughout the data collection to ensure replicability. Anthropometric data and personal data were collected immediately after inclusion. Standardized warm-ups were performed before force and sprint assessments. With the agreement of their coaches, participants were asked not to sprint or train intensively during the 72h preceding the tests to avoid muscle damage or fatigue. For the sake of space, the steps are summarized below, and additional procedural details are provided in Supplemental Online Material 1.

#### Questionnaires

Several personal, psychological, and behavioural health-related risk factors were collected online using tablets and LimeSurvey software (v3.17.3, LimeSurvey GmbH, Hamburg, Germany). Questionnaires were split into 4 groups (mean completion time: 3.8 − 4.8 min/group). The evaluated risk factors and their associated questionnaires are presented in Table 1. Further details about the questionnaires are available in Supplemental Online Materials 1 and 2.

**Table 1.** Questionnaires used in the present study and their grouping.

Risk factors	Questionnaires
Group A	
Athletic identity	Athlete Identity Measurement Scale (AIMS) [29,30], 10 items
Nutritional habits	One-item Self-rated diet [31] Usual junk food intake [31], 6 items French version of the Sick, Control, One, Fat, Food scale (SCOFF-F) [32,33], 5 items
Inclination to play through pain	One-item question about susceptibility to persist through pain [34] Subjective norms in soccer, 3 items based on Ajzen (2006) [35] recommendations
Perceived health knowledge	One-item adapted from Sorg et al. (2020) [36]
Perceived susceptibility to soccer-related injury	Perceived Susceptibility to Sport Injury scale (PSSI) [34,37] adapted for soccer, 4 items
Group B	
Achievement Goals	French Achievement Goals Questionnaire for Sport and Exercise (FAGQSE) [38], 12 items
Sport Anxiety	Modified Sport Anxiety Scale (SAS) [39], 16 items
Sleep quality	French version of the Athens Insomnia scale (AIS-FR) [40], 8 items
Sleep quantity	Single item about usual sleep duration (from the Pittsburgh Sleep Quality Index) [41]
Group C	
Coping strategies	Ways of Coping Checklist (WCC) [42], 27 items
Personality	10-item version of the Big Five Inventory (BFI-10) [43]
Group D	
Sleep behaviour	The French version of the Athlete Sleep Behaviour Questionnaire (ASBQ-FR) [40], 15 items
Burnout	Athlete Burnout Questionnaire (ABO-S) [44], 15 items

#### Maximal voluntary isometric contractions

For each force/torque measurement, all participants had visual feedback of their force/torque and standardized strong encouragements were provided. Lower limbs forces/torque were assessed unilaterally, and at least 2 trials were recorded for each lower limb and position (see Supplementary Figure 1 and Supplementary Table 1 for further details). If the difference in peak force values between the 2 trials exceeded 5%, a third maximal contraction was performed. One minute of rest was taken between 2 trials. The best performance among the trials was retained.

Maximal knee extensors and flexors torques were assessed by conducting maximal voluntary isometric contractions (MVICs) on specific ergometers (S2P, Science to Practice, Ljubljana, Slovenia and Hamtech, Human Kinematic, Carros, France). Knee extensors contractions were performed at 80° of knee and hip flexion (0° = anatomical position reference). Knee flexors contractions were performed in 3 different positions: long hamstring muscle length (LL), close to the usual injury position during sprints during the late swing phase (30° of knee flexion and 50° of hip flexion); intermediate hamstring muscle length (IL) (30° of knee flexion and 0° of hip flexion); and short hamstring muscle length (SL) (80° of knee flexion and 0° of hip flexion).

#### Repeated sprints

Participants performed 10 repetitions of 30-m all-out sprints on a synthetic pitch, starting from a static standing position every 30s (see Supplementary Figure 2 and Supplementary Table 2 for further details). Sprint times were recorded using photocells (Witty; Microgate Srl, Bolzano, Italia). The fastest sprint time achieved (Best Sprint) and the repeated sprint ability index (RSA_index_; Equation 1) were retained for the analysis [45].

(1) $${\text{RSA}}_{\text{Index}}(\%)={((\text{Sprint}}_{\text{TT}}-{\text{ideal Sprint}}_{\text{TT}})/{\text{ideal Sprint}}_{\text{TT}})\times 100$$

Where Sprint_TT_ is the sprint total time calculated as the sum of the times for all 10 sprints and ideal Sprint_TT_ is the Sprint_TT_ if all the sprints were run at the best time.

The maximal torques of the knee flexors in the LL position were reassessed after the RSA test (1 min 19s ± 29s). Knee flexors performance fatigability was quantified as the percentage change from pre- to post-RSA test. Before and after the RSA test, participants also had to rate their perceived fatigue, defined as the alteration of the capacity to produce a maximal effort [46], on a 10-point numerical scale [47].

Horizontal velocity was recorded at 46.875 Hz by a radar (Stalker Pro II Sports Radar Gun; Plano, TX, USA) during each sprint [48]. The radar was positioned to point at the center of mass (at 1-m from the floor) and 10-m behind the starting point of the sprints. Horizontal velocity data were processed using the methodology outlined by Samozino et al. [49] to obtain an individual force-velocity (F-V) profile for each sprint (Supplementary Figure 3). The maximum value of the theoretical maximal horizontal force (max F_0_) and velocity (max V_0_), and the maximum value of the maximal mechanical power (max Pmax) were retained for analysis (Supplementary Table 3). We also calculated the changes in F_0_, V_0_ and Pmax observed during the RSA test.

Sprint patterns were observed during the acceleration phase (at 5 m) and the maximal velocity phase (at 25 m) of each sprint. Cameras 240 Hz (Ipad Pro, Apple Inc., Cupertino, CA, USA) recorded the running kinetics in the sagittal and frontal planes. Videos were edited to select the late swing phase of the most centered step (from contralateral touchdown to homolateral touchdown), for each sprint. Joint positions were estimated using OpenPose [50] scripts (v1.7.0), a markerless deep learning method (Supplementary Figure 4). In the sagittal plane, our variable of interest was the peak angle θS. θS was calculated by subtracting the homolateral knee flexion angle and contralateral hip flexion angle from the homolateral hip flexion angle (Supplementary Table 4). This variable represents the maximal length of the hamstring during the late swing phase (adapted from Wilmes et al. [51]. In the frontal plane, our variable of interest was the angle between the shoulders and the pelvis (θF) to represent the frontal thoraco-pelvic control during the sprint.

We retained the following values, measured at 5 m and 25 m, as parameters for the analysis: the angles in pre-fatigue and fatigue conditions, the maximal angle values during the RSA test, and the changes in these angles with fatigue.

### Injury reporting

During the 10-month follow-up, injuries were self-reported by the participants every month through a questionnaire sent by email. To ensure compliance and accuracy, these reports were cross-checked with the assistance of physical trainers or medical staff from each team. Hamstring strain injury was defined as an acute pain in the hamstrings location that occurred during training or competition and resulted in the immediate termination of play or inability to participate in the next training session or match [21]. Injury type, side and location, date of occurrence, and date of return to full training with the group were recorded [52]. HSI severity was defined as the number of days that elapsed from the date of injury to the date of the player’s return to full participation in team training and availability for match selection [53], and classified as slight (0 days), mild (1–7 days), moderate (8–28 days), or severe (>28 days) [2,52]. Only initial HSI were considered for the analysis.

### Statistical analysis

#### Data preprocessing and descriptive analyses

Following current data preprocessing recommendations [54], we cleaned our data before further analysis. Missing data were treated with the Hot Deck imputation method [55], and data were normalized using Z-score normalization to have mean  = 0 and standard deviation  = 1 for each continuous variable [20,54]. Mean (M), standard deviation (SD), skewness and Kurtosis were calculated for all continuous variables. Data were considered to follow a normal distribution if skewness was ≤2 and kurtosis was ≤7. Correlations were analyzed using Spearman coefficients.

#### Variable selection

To avoid bias linked to the consideration of too many variables, we proceeded to a variables’ selection. First, we analyzed the variance equality using Fisher test. Then, we tested the group differences between participants who sustained at least 1 HSI during follow-up and those who did not by conducting a T-test for 2 independent samples or a Mann Whitney U-test, depending on the Fisher’s test result and the distribution of the data. To avoid excluding relevant variables, we included in the logistic regression analysis all independent variables with *p*≤.10 and also well-recognized HSI risk factors (i.e. age, HSI history and sex) [56,57], regardless of their univariate results. To address multicollinearity, we excluded independent variables with significant correlations higher than .70 (*p*<.05) and redundant variables (i.e. computation from a same measurement), only retaining the most theoretically pertinent variables. Logit linearity was verified using preliminary Box-Tidwell transformation. For the categorical variables, we defined as ‘1’ the female sex and ‘0’ the male sex, and ‘1’ the presence of HSI history and ‘0’ the absence of HSI history.

#### Logistic regressions

Binary logistic regressions were conducted using the Wald backward stepwise method until all the individual variables of the model reached *p*≤.10. At each step of the analysis, the overall model fit was assessed with Hosmer-Lemeshow goodness-of-fit tests (i.e. *p*≥.05 and the smallest chi-square possible), and the Nagelkerke R-square index (the higher, the better). The significance of individual variables was assessed through the Beta coefficient (B), the Wald index significance level (*p*≤.05), the exponential of Beta representing the odds ratios (ORs), and their corresponding 95% confidence intervals [40]. For internal validation of the model, 200-iterations bootstrapped resampling was used. The coefficients of the bootstrapped model were compared to the initial model and adjusted. Calibration was verified by plotting the data in deciles and comparing the agreement between the model’s predicted risk of HSI and the observed proportion of HSI in each decile. Plotted data were reported with their corresponding 95% confidence intervals. A perfect prediction lies on the reference line (i.e. intercept  =  0 and slope  =  1) [58]. Discrimination was assessed by the percentage of correct classifications of the injury status of the players by the model. Given the expected oversampling of uninjured athletes (∼84% uninjured versus 16% injured) and the preventive perspective of this study, we aimed to achieve a balance between sensitivity and specificity rather than striving for the highest overall correct prediction percentage. The cut-off point was defined using receiver operating characteristic (ROC) curves to maximize both sensibility and specificity.

AUC of the ROC curves were used to evaluate the accuracy of the discrimination of the predictive model (.5 = equivalent to random chance; 1.0 = perfect prediction) and to compare the predictive performance of the model versus the independent variables individually [20].

## Results

One hundred and twenty-two participants ultimately took part in this study (22 females and 100 males) (Table 2). Twenty-four of them presented a history of HSI. Two participants were excluded because of a pain in their knees. Missing data accounted for 3.7% of our total data collection (Supplemental Online Material 3). They resulted from experimenter errors (i.e. confusion in questionnaire group attribution), participant mistakes (i.e. participants kicking the sensor during post-sprint torque measurements), technical failures (i.e. radar dysfunction during the repeated sprints), or data transfer failures (i.e. automatic cutting of the first or last sprints during some video data transfers). The independent variables were individually imputed between 0% and 3.3%, except for running kinematics variables (9.0%) and force-velocity profile variables (14.2%).

**Table 2.** Personal characteristics of the players (N = 120).

CHARACTERISTICS	MEAN ± SD OR NUMBER
Age	18.3 ± 3.5 years old
Body mass	66.6 ± 9.0 kg
Height	1.75±.08 m
Profession	
High school student	65
University student	23
Professional soccer player	18
Worker	14
Dominant lower limb	
Right	87
Left	33
Playing position	
Forwards	27
Defenders	28
Wingers	22
Midfielders	43
Playing level	
Regional	16
National	98
International	6
Experience in soccer	11.6 ± 3.6 years
Training time per week	9.9 ± 3.8 hours

*Abbreviations:* m = meters; kg = kilograms

### Epidemiology of hamstring strain injuries

Twenty-nine participants, representing 24% of our population, sustained at least 1 HSI during the follow-up period. The severity of index injuries was mild (*N* = 10), moderate (*N* = 17), or severe (*N* = 2). Among the initial injuries, 10 occurred during the preseason or early season (August-September), 12 occurred during the middle of the competitive season (from October to March), and 7 HSIs occurred at the end of the competitive season (April–May). The distribution of HSIs between the legs was 56% to the dominant leg and 44% to the non-dominant leg.

### Logistic regressions

#### Variable selection

Eight variables showed group differences between players who sustained a hamstring injury and players who were uninjured (see Supplemental Online Material 2). The independent variables selected following a comparison of the groups are presented in Table 3.

**Table 3.** Means, standard deviations and group differences between injured and uninjured players.

Prospective follow-up	Groups		Mean difference between groups	
	Uninjured	Injured	*p*-value (T-test)	*p*-value (U-test of Mann-Whitney)
Sex	16♀ (18%) / 75♂ (82%)	6♀ (21%) / 23♂ (79%)		
HSI history	14 (17%)	10 (34%)		
Age (years old)	17.8 ± 2.6	19.7 ± 5.1		.106
Subjective norms (/21)	13.9 ± 4.2	15.4 ± 3.3	.085	
Perceived vulnerability (/28)	12.5 ± 3.6	14.3 ± 4.8	.072	
RSA_Index_ (%)	6.9 ± 3.8	8.8 ± 6.4		.055
Best sprint (s)	4.4±.2	4.3±.2	.036	
Tmax_KF_ at SL Dom (N.m)	118.8 ± 30.4	132.9 ± 39.5	.045	
Tmax_KF_ at LL Dom (N.m)	143.4 ± 33.2	157.1 ± 40.8	.070	
ΔTmax_KF_ in Post1 Dom (%)	−4.8 ± 12.3	−11.7 ± 11.2	.008	
Max V_0_ (m.s^-1^)	8.8±.7	9.1±.8	.035	

♀ = female; ♂ = male; % = percentage.

*Abbreviations:* ΔTmax_KF_ in Post1 Dom = percentage loss between pre-sprint and the first measure post-sprint of the isometric torque of knee flexors at long muscle length; HSI = Hamstring strain injury; m = meters; Max V_0_ =  maximal value over all the sprints of the theoretical maximal velocity; N = newtons; RSA_Index_  =  percentage difference between the ideal time and the split total time; s = seconds; SD = Standard Deviation; Tmax_KF_ at LL Dom = Maximal isometric torque of knee flexors at long muscle length; Tmax_KF_ at SL Dom = Maximal isometric torque of knee flexors of the dominant leg at short muscle length.

The correlation tests between the previously selected variables showed a high significative bilateral correlation only between Tmax_KF_ at SL Dom and Tmax_KF_ at LL Dom (*r* = .81, *p*<.001). Furthermore, ΔTmax_KF_ in Post1 Dom was computed from Tmax_KF_ at LL Dom. Thus, to avoid multicollinearity and given the usual context in which HSIs occur (i.e. in fatigue conditions), we excluded Tmax_KF_ at SL Dom and Tmax_KF_ at LL Dom from our logistic regression model. We retained only ΔTmax_KF_ in Post1 Dom as an independent variable.

To respect the logit linearity assumption, perceived vulnerability was squared, renormalized and renamed ‘squared perceived vulnerability’. Then, logit linearity was verified for all the independent variables.

#### Logistic regression model selection and internal validation

**Figure 2.**
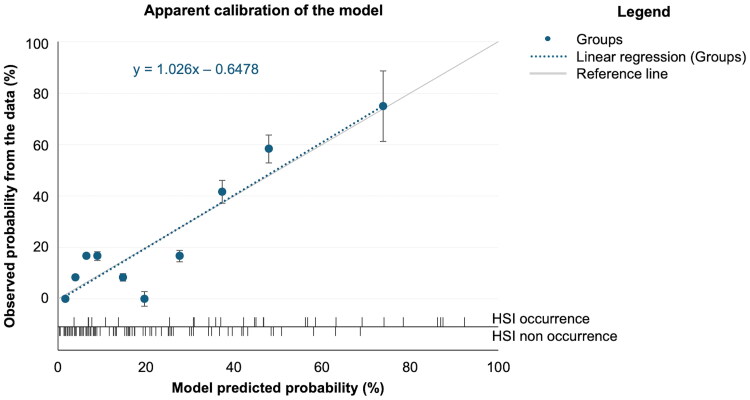
Apparent calibration of the final predictive model (without adjustment for overfitting). Group data are presented with their 95% confidence intervals. HSI = hamstring strain injury.

The value of using a model was confirmed by the rejection of the null hypothesis (*p*<.001). The results of the Wald backward stepwise analysis are presented in Table 4. The Nagelkerke R-square index (.357, .356, .343, and .332 for steps 1, 2, 3 and 4, respectively) and the Hosmer-Lemeshow chi-square (Table 4) showed a good adjustment for the different models (*p*>.05). We selected the step 2 model, which presented the best overall fitting indexes and thus offered the best replicability (Hosmer-Lemeshow chi-square  =  8.413, *p* = .394). The internal validity of the model was confirmed by identical regression coefficients in the bootstrapped model. The model presented good calibration, with a slightly negative intercept (intercept =  −0.65) and an almost perfect slope (slope  =  1.03) (Figure 2).

**Table 4.** Successive models of the Wald backward stepwise analysis.

	Hosmer and Lemeshow test							ORs 95% confidence interval	
	Chi-square	*p*-value	B	B(SE)	Wald	*p*-value	ORs	Inferior	Superior
Step 1	8.499	.386							
Sex			2.406	1.220	3.888	.049*	11.095	1.014	121.340
Age			.368	.239	2.369	.124	1.444	.904	2.307
HSI history			1.026	.595	2.979	**.084**	2.791	.870	8.952
Best sprint			−.862	.476	3.272	**.070**	.422	.166	1.075
ΔTmax_KF_ in Post1 Dom			−.753	.295	6.536	.011*	.471	.264	.839
Subjective norms			.361	.309	1.366	.242	1.435	.783	2.631
RSA_Index_			−.079	.299	.069	.792	.924	.515	1.659
Max V_0_			.369	.302	1.491	.222	1.446	.800	2.615
Squared perceived vulnerability			.423	.238	3.153	**.076**	1.527	.957	2.437
Constant			−2.266	.468	23.478	**<**.001*	.104		
Step 2	8.413	.394							
Sex			2.264	1.085	4.349	.037*	9.619	1.146	80.741
Age			.359	.237	2.308	.129	1.432	.901	2.277
HSI history			1.034	.594	3.034	**.082**	2.813	.879	9.010
Best sprint			−.823	.452	3.314	**.069**	.439	.181	1.065
ΔTmax_KF_ in Post1 Dom			−.735	.287	6.579	.010*	.479	.273	0.841
Subjective norms			.359	.310	1.343	.246	1.432	.780	2.627
Max V_0_			.360	.300	1.444	.229	1.434	.797	2.580
Squared perceived vulnerability			.421	.238	3.124	**.077**	1.524	.955	2.431
Constant			−2.234	.448	24.847	**<**.001*	.107		
Step 3	12.386	.135							
Sex			2.124	1.053	4.069	.044*	8.365	1.062	65.885
Age			.414	.232	3.186	**.074**	1.514	.960	2.386
HSI history			1.087	.593	3.354	**.067**	2.965	.927	9.486
Best sprint			−.853	.444	3.682	**.055**	.426	.178	1.018
ΔTmax_KF_ in Post1 Dom			−.677	.279	5.913	.015*	.508	.294	.877
Max V_0_			.311	.294	1.122	.290	1.365	.768	2.426
Squared perceived vulnerability			.483	.232	4.343	.037*	1.621	1.029	2.552
Constant			−2.191	.437	25.161	**<**.001*	.112		
Step 4	11.562	.172							
Sex			2.157	1.048	4.235	.040*	8.642	1.108	67.389
Age			.417	.225	3.437	**.064**	1.518	.976	2.360
HSI history			1.013	.589	2.964	**.085**	2.755	.869	8.733
Best sprint			−1.021	.419	5.953	.015*	.360	.159	.818
ΔTmax_KF_ in Post1 Dom			−.683	.276	6.111	.013*	.505	.294	.868
Squared perceived vulnerability			.473	.231	4.198	.040*	1.606	1.021	2.525
Constant			−2.157	.429	25.310	**<**.001*	.116		

*p*-value in bold = *p*<.10; * = *p*<.05; framed model = final selected model.

*Abbreviations:* B = Beta coefficient; B(SE) = standard error; ΔTmax_KF_ in Post1 Dom = percentage loss between pre-sprint and the first measure post-sprint of the isometric torque of knee flexors of the dominant lower limb at long muscle length; HSI = Hamstring strain injury; Max V_0_ = maximal value of the theoretical maximal velocity of the 10 sprints; ORs = ratio-change in the odds of the event ‘injury to the hamstring’ for a one-unit change in the predictor; RSA_Index_ = percentage difference between the ideal time and the split total time.

#### Cut-off point definition and discrimination

Using the ROC curve, a cut-off point to classify the players as at risk of HSI was defined at .253. This resulted in sensibility (i.e. correct classification of the injured players by the model) of 79.3%, specificity (i.e. correct classification of the uninjured players) of 75.6%, and a global correct classification of 76.7%. The model presented a very good predictive performance (*p*<.001, AUC = .815, 95%CI =  [.719–.912]).

The conditional probability of a player getting injured [P(Injured)] was calculated using Equation 2 [41].

(2) $$P(\text{Injured})=e^{g(x)}/\left(1+e^{g(x)}\right)\;$$

With

(3) $$g(x)=(2.264\times \text{sex})+(0.359\times \text{age})+(1.034\times \text{HSI history})–(0.823\times \text{Best sprint})–(0.735\times {\text{ΔTmax}}_{\text{KF}}\text{ in Post}1\;\text{dom})+(0.369\times \text{Subjective norms})+(0.360\times \text{Max}\;V_{0})+(0.421\times \text{Squared perceived vulnerability})–2.234$$

Where, female sex  =  1 (male  =  0), and the presence of an HSI history  =  1 (absence of HSI history  =  0). All the variable scores are normalized in Z-score.

#### Comparison of the predictive performance of the model and the variables considered independently

**Figure 3.**
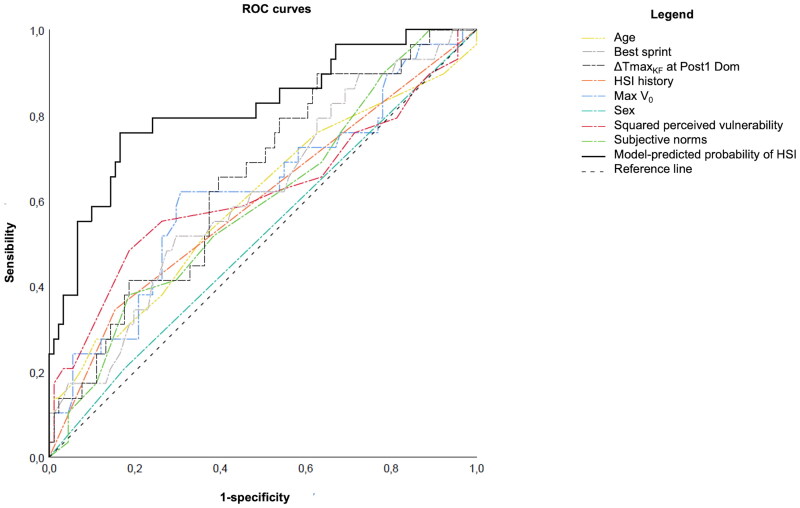
Receiver operating characteristic curves of the model and of each individual variable of the model when considered separately. Abbreviations: ΔTmax_KF_ in Post1 dom = percentage loss between pre-sprint and the first measure post-sprint of the isometric torque of knee flexors of the dominant lower limb at long muscle length; HSI = hamstring strain injury; Max V_0_ = maximal value of the theoretical maximal velocity of the 10 sprints; ROC curves = receiver operating characteristic curves.

Among the individual predictors, only %ΔTmax_KF_ in Post1 Dom presented marginal predictive performance (*p* = .016, AUC = .649, 95%CI = [.538–.761]). Max V_0_ (*p* = .051, AUC = .621, 95%CI = [.498–.744]) and perceived vulnerability (*p* = .057, AUC = .618, 95%CI = [.484–.752] were slightly above the significance threshold of .05. None of the other variables of the model presented predictive performance individually (age: *p* = .113, AUC = .598, 95%IC = [.472–.724]; subjective norms: *p* = .123, AUC = .595, 95%CI = [.477–.713]; injury history: *p* = .122, AUC = .595, 95%CI = [.471–.720], and sex: *p* = .802, AUC = .516, 95%CI = [.393–.638]). ROC-curves are presented in Figure 3.

## Discussion

Using a multifactorial approach, we developed an encouraging preliminary model (AUC = .815) to identify soccer players at risk of HSI, achieving a sensitivity of ∼80% with only eight predictors. The present study is a pioneer in HSI predictive research by considering a wide range of HSI risk factors, including physiological, psychological, kinetic, kinematic, performance fatigability and personal risk factors as predictors. This model is applicable to both professional and amateur soccer players, affirming the importance of considering injury predictors together, rather than evaluating them separately.

In this exploratory study, our 8-variable logistic regression model explained 36% of the variance in HSI occurrence and accurately predicted 79% of the players who sustained an HSI in our sample. Compared to other predictive models for HSI occurrence based on the preseason screening of players, our model shows better performance than Ruddy et al. [20] within-year predictive models (median AUC<.60), and equivalent performance to Ayala et al.’s [21] ADtree model (AUC = .84). Given the multifactorial etiology of HSI, Ruddy et al.’s [20] model may have been limited by the relatively small number of different predictors considered. In contrast, Ayala et al. [21] considered a wide range of predictors, resulting in an efficient model. However, their model required the measurement of 66 variables, making it complex for field use, and it did not include some ecologically relevant measurements (i.e. related to sprint, performance fatigability or psychological characteristics). One of the strengths of the present preliminary model is also to take into consideration relevant and innovative predictors such as hamstring performance fatigability (i.e. the decrease of the peak torque of the knee flexors following repeated sprints), sprint performance parameters (i.e. maximal V_0_ and best sprint), psychological variables (i.e. perceived vulnerability and subjective norms in soccer) and personal characteristics (i.e. HSI history, sex and age). Our preliminary model allows us to include a reasonable number of theoretically relevant variables, while providing performance comparable to more complex models. This ease of use and efficiency make it a promising tool for predicting injury risk in practical field applications.

Furthermore, our study produced a significant original finding, highlighting knee flexors performance fatigability following repeated sprints as a crucial predictor for HSI occurrence. Until now, fatigue has only been assumed to be a risk factor for HSI due to its impact on intrinsic risk factors such as the decrease of hamstring strength, alteration of sprint patterns, and deterioration of muscle coordination [59]. In addition, the large proportion of HSIs occurring towards the end of halves in soccer matches has further emphasized the significance of fatigue in injury risk [5]. Our experimental findings show that performance fatigability itself is a predictor of HSI occurrence. Specifically, the likelihood of sustaining an HSI is reduced by more than half for every standardized unit decrease of ΔTmax_KF_ at Post1, if all other parameters remain stable (ORs = .48, 95%CI [.27–0.84], *p* = .01).

In the present study, the RSA-test induced knee flexor performance fatigability comparable to that reported by Franceschi et al. [60] after a soccer match (respectively −9.0% and −10.0% for the dominant lower limb). Thus, ΔTmax_KF_ in Post1 emerged as an ecological and valuable performance fatigability marker for predicting HSI occurrence. It is also noteworthy that pre-sprint isometric peak torques tend to be higher in the injured group than in the uninjured group (*p* = .070 for Tmax_KF_ at LL, *p* = .124 at ML, and *p* = .045 at SL), which aligns with conflicting findings on hamstring peak torque as a potential risk factor for HSI [8,15]. In addition, post-sprint torques were similar in both groups (*p* = .80 for Tmax_KF_ in Post1 in both lower limbs), challenging the hypothesis of a universal lower threshold of hamstring force that renders soccer players more susceptible to strains [7]. While these findings need to be confirm using causal inference approaches, they suggest that the decrease in hamstring torque might be more critical for preventing HSI compared to initial torque.

Furthermore, hamstring performance fatigability seems more adapted than maximal horizontal force production decreases (ΔF_0_) in identifying future HSIs. Since F_0_ reflects a combination of posterior chain muscles (e.g. gluteus major, hamstring, gastrocnemius), compensatory strategies may hide a hamstring weak point, highlighted by hamstring torque decrement (ΔTmax_KF_ in Post1) measurement. Future research using electromyography and inverse dynamics analysis during sprinting could provide deeper insight into fatigue-induced adaptations and their potential role in HSI risk.

Our predictive model also identified predictors related to sprint performance. Players with faster sprint times (i.e. smaller ‘Best sprint’ time), and those with higher V_0_ appeared to be more susceptible to sustaining an HSI (ORs_Best sprint_ = .44, 95%CI [.18–1.07], *p* = .07; ORs_Max V0_ = 1.43, 95%CI [.80–2.58], *p* = .23). This is not the first time that sprint performance has been associated with soccer injuries. Faster soccer players on the 40 m sprint present higher groin injury rates [61], and prospectively injured soccer players tend to be the fastest over 20 meters sprint on preseason screening (*p* = .052) [62]. Although Max V_0_ did not reach significance at *p*<.10 in our model, theoretically faster players have higher lower-limb angular velocity [63], which in turn increases the strain on the hamstrings during the late swing phase of each stride. Moreover, under conditions of fatigue, the hamstrings may be too weak to withstand this heightened strain, thereby contributing to the occurrence of HSIs.

Another strength of our model is the consideration of various psychological variables. Among the wide range of psychological risk factors for sport injury considered in this predictive model, perceived vulnerability to soccer-related injuries was the most relevant predictor regarding HSI occurrence (ORs  =  1.5, 95%CI [.96–2.43], *p* = .08 in our predictive model). As discussed in Chalabaev et al. [34] this perceived vulnerability could result from a controlled motivation with the mediation of perceived susceptibility to persist through pain. It could be an indicator of the contrast between physical vulnerability due to excessive load (e.g. fatigue, pain) or resulting from injury or re-injury anxiety [64], and playing demands [14]. Unsurprisingly, internalization of subjective norms regarding pain and fatigue in soccer were also an HSI predictor in the model, although not significant (ORs_subjective norms_ = 1.43, 95%CI [.78–2.63], *p* = .25). The internalization of these subjective norms, akin to the ‘no rest culture’ (also named ‘pain norm’ or ‘socialization’) [64,65], can lead to ignoring body signals or persisting through pain, which have been identified as situational risk factors of HSI [13,14]. Results of the present study were in line with Baize et al.’s [14] findings, where soccer players continued to play despite pain because of the love of game and the fear of negative staff evaluation, and exceeded their limits before getting injured, regardless of whether they felt vulnerable or not. Within the framework of the stress injury model [66], the perception of vulnerability and the internalization of norms could potentially lead to perceiving playing as a threat, thereby increasing the risk of HSI. It can be hypothesized that this may result in a psychological and physical mismatch, like the willingness to overcome bodily fatigue and limits, that amplifies the disparity between, on the one hand, the muscles’ demands and maintaining the intensity of the game and, on the other hand, the capacity for force production, all exacerbated by fatigue. Further research is needed to fully explore this hypothesis.

In our study, we confirmed that personal characteristics (i.e. sex, age and HSI history) are important non-modifiable predictors of HSI. Contrary to prior findings [67,68], female soccer players had a 9.6 times higher probability of getting injured than males in our model (ORs_sex_ = 9.61, 95%CI [1.15–80.74], *p* = .04), with other parameters being constant. These results could be explained by a mathematical compensation of the sex difference in sprint and fatigue performance variables. It could also be attributed to the relatively small number of females in our sample (22 players, 20% of our sample), which is a limitation in our model, and their high injury rate compared to previous studies. In line with prior findings, HSI history increased the prospective HSI risk by 2.8 times (ORs_HSI history_ = 2.81, 95%CI [.88–9.01], *p* = .08). Regarding age, each standardized unit increase in age increased the risk of HSI by 1.4 times, although this contribution was not statistically significant in the model (ORs_age_ = 1.43, 95%CI [.90–2.28], *p* = .13). The relatively young and homogeneous population (70% of our sample was between 16 and 19 years old) might explain the lesser impact of age in our model compared to previous studies [69].

As a result, predicting HSI occurrence using the present preliminary model requires the assessment eight predictors. While it might be tempting to measure only 1 or 2 parameters in the field, the study revealed that none of the predictors measured, considering individually, displayed good predictive performance. Only ΔTmax_KE_ in Post1 performed significantly better than chance, with a low AUC = .65, whereas the model presented a very good predictive capacity (AUC = .82). These results corroborate those of Opar et al. [70] who found low AUC (≤.64) for predictors evaluated independently compared to their model (AUC = .681 with preseason screening, AUC = .726 with multiple time points), in Australian football.

Although these are preliminary results, this study offers a straightforward approach to identify players susceptible to HSI through a single screening session. Recognizing that time constraints are a prevalent obstacle to implementing preventive strategies [71,72], we ensured that the proposed method seamlessly integrates into preseason preparations and overall screening processes. The screening only requires 2 psychological questionnaires and 1 RSA test with kinematic measures with hamstring force assessment before and after the test. To facilitate the final identification of players at risk of HSI with the regression model, an excel table is provided in Supplemental Online Material 4. It can be completed with unstandardized data. Knowing their individual risk, players can better adhere to preventive exercise.

### Limitations

Despite the rigorous execution of this study, it is essential to acknowledge several limitations. While the present model has good fit indexes, it requires an external validation in a separate sample to confirm its predictive power. Models trained on one sample may demonstrate overfitting on another sample [56]. Additionally, to enhance its applicability, external validation could be conducted across more various player skill levels, age groups, or even different sports. As a future perspective, applying our model to HSI prevention by identifying at-risk players and implementing targeted prevention programs could be compared to existing injury prevention methods to assess its added value or limitations.

Moreover, event per predictor recommendations have not been reached with our final sample size [25]. Although a posteriori sample size calculation confirmed sufficient power to ensure a shrinkage ≥.80, the sample size did not ensure the precise estimate of overall risk (model intercept) [25]. The small sample size could lead to overfitting of the model. The proportion of imputations among Max V_0_ variable could also bias the results of this variable, representing a limitation. Furthermore, the scant representation of females may reduce the generalizability of the results within this population. Given that young male soccer players constitute most of our sample, this preliminary predictive model is likely more applicable to them. To enhance its validity and applicability across different populations, future studies should include larger sample size with better representation of females and older players.

Although all participants were tested within a relatively short timeframe of approximately six weeks (July 26–September 9, 2022), variations in training intensity between teams and cultural differences may have influenced the test results. Furthermore, with a unique measurement session, we assumed stability in the predictors. We took care to select questionnaires assessing players’ dispositions rather than their psychological states during the screening period. However, variations in extrinsic risk factors, including training load, time of the season, and coaching style [5] throughout the soccer season may impact predictors related to sprint performance, maximal force performance, and performance fatigability. Measuring these variations over the soccer season and observing potential relationships could provide valuable insights. Nevertheless, Opar et al. [55] reported that incorporating multiple time points during the season did not improve the ability to predict injury risk. Therefore, while including extrinsic risk factors and monitoring predictors over time may modify our model, it may not necessarily enhance its overall performance.

Finally, while our model incorporates multiple predictors from different domains, it does not account for potential interactions between them. Injury risk is inherently multifactorial, and interactions may exist between physiological, psychological, and performance-related predictors. Although we mitigated multicollinearity by excluding highly correlated variables [41], interactions between predictors may still exist and influence injury risk. Future research should investigate these interactions in a larger population using statistical approaches, such as multilevel modeling, Bayesian modeling or the web of determinants [73], to further refine and enhance the predictive model.

## Conclusions

While assessing a single predictor does not provide an accurate prediction of HSI risk among soccer players, our multidisciplinary approach, considering diverse risk factor categories as predictors, demonstrates promising predictive performance. This model integrates several predictors, including personal characteristics (age, sex and HSI history), knee flexors performance fatigability following repeated sprints, sprint performance (sprint time and V_0_), and psychological variables (perceived vulnerability to injuries and the assimilation of subjective norms in soccer). However, this model remains preliminary and requires further validation in independent cohorts to confirm its robustness and applicability. The present study provides practitioners with a preliminary version of an integrative and parsimonious model to help them in identifying players at risk of HSI through a short preseason screening.

## Supplementary Material

Supplemental Material

## Data Availability

The data that support the findings of this study are available on request from the corresponding author, Enzo Piponnier. The data are not publicly available due to the sensitive nature of the research and French laws protecting the privacy of health-related information.

## Submission statement

All authors have read and agree with the manuscript content. This article has not been published previously and is not under consideration for publication elsewhere. While this manuscript is being reviewed for this journal, it will not be submitted elsewhere for review and publication. Its publication is approved by all authors and tacitly or explicitly by the responsible authorities where the work was carried out, and, if accepted, it will not be published elsewhere including electronically in the same form, in English or in any other language, without the written consent of the copyright-holder.
