# Supplementary material for: Development of a preliminary multivariable model predicting hamstring strain injuries during preseason screening in soccer players: a multidisciplinary approach
Source: Ann Med. 2025 May 8;57(1):2494683. doi: 10.1080/07853890.2025.2494683 (PMC12064112; doi:10.1080/07853890.2025.2494683)
Supplement: Supplemental Material [file IANN_A_2494683_SM9750.zip › suppl_data/Supp1 Figure caption.docx]

Supplementary Fig. 1. Position for the maximal voluntary isometric contraction of A) knee extensors, B) knee flexors at short hamstring muscle length, C) knee flexors at intermediate hamstring muscle length and D) knee flexors at long hamstring muscle length.

Supplementary Fig. 2. Placement of the measurement material in the RSA test.

Supplementary Fig. 3. Illustration of the curves obtained from radar data and the calculated Force-Velocity and Power-Velocity profiles. A. Example of the horizontal velocity and acceleration curves as function of time, obtained by radar and modeling. B. Example of force-velocity and power-velocity curves.

Supplementary Fig. 4. OpenPose Body_25 keypoints model during the repeated sprints and angles of interest (A) in the sagittal view and (B) in the frontal view.
