# Supplementary material for: Development of a preliminary multivariable model predicting hamstring strain injuries during preseason screening in soccer players: a multidisciplinary approach
Source: Ann Med. 2025 May 8;57(1):2494683. doi: 10.1080/07853890.2025.2494683 (PMC12064112; doi:10.1080/07853890.2025.2494683)
Supplement: Supplemental Material [file IANN_A_2494683_SM9750.zip › suppl_data/Supplemental online material 1 Tables.docx]

# Supplemental online material 1 - Tables

Table 1. Torque independent variables

| **Variables (per lower limb)** |
| --- |
| Knee extensors maximal voluntary isometric contraction torque (Tmax_KE_; N.m) |
| Knee flexors maximal voluntary isometric contraction torque (Tmax_KF_; N.m)   - At long hamstring muscle length (Tmax_KF_ at LL) - At intermediate hamstring muscle length (Tmax_KF_ at IL) - At short hamstring muscle length (Tmax_KF_ at SL) |
| Torque imbalance between the dominant and the non-dominant lower limb (imb; Equation SF3-1)   - For knee extensors (imb_KE_; %) - For knee extensors at LL (imb_KF_ at LL; %) - For knee extensors at IL (imb_KF_ at IL; %) - For knee extensors at SL (imb_KF_ at SL; %)   This was calculated as:  (Equation SF3-1) $imb=ABS[(Tmax Dom-Tmax Non dom)\times100/Tmax Dom]$  Where ABS is the absolute value, Tmax is the related maximal peak torque, Dom is the dominant lower limb, and Non dom is the non-dominant lower limb. |

Abbreviations: IL=intermediate hamstring muscle length, imb=imbalance between the dominant and the non-dominant lower limb, KE=knee extensors, KF=knee flexors, LL= long hamstring muscle length, m=meters, N=newtons, Post1=first force/torque measurement post-sprints, Post2=second force/torque measurement post-sprints, SL=short hamstring muscle length, Tmax=maximal peak torque.

Table 2. Independent variables related to repeated sprint ability.

| **RSA variables** |
| --- |
| Fastest sprint time achieved (Best sprint; s) |
| Repeated sprint ability index (RSA_index_; %), representing the percentage of decrease of the sprint performance during the repeated sprints, calculated as Eq. (D.1):  Eq. (1) ${RSA}_{Index}(\%)={{(Sprint}_{TT}-{ideal Sprint}_{TT})}/{{ideal Sprint}_{TT}}\times100$  Where Sprint_TT_ is the sprint total time calculated as the sum of the times for all ten sprints and ideal Sprint_TT_ is the Sprint_TT_ if all the sprints were run at the best time (i.e., ideal Sprint_TT_=10xBest sprint). |
| Pre- to post-sprints absolute change in the perceived fatigue (ΔPerceived fatigue; rate from 0 to 10)^20^ |
| Knee flexors maximal voluntary isometric contraction torque at LL after RSA test   - In the first trial (Tmax_KF_ in Post1; N.m) - In the second trial (Tmax_KF_ in Post2; N.m) |
| Knee flexors performance fatigability calculated as the percentage difference in Tmax_KF_ at LL between pre- and post-RSA test   - In the first trial (ΔTmax_KF_ in Post1; %) - In the second trial (ΔTmax_KF_ in Post2; %) |

Table 3. Independent variables related to the force-velocity profiles of the repeated sprints.

| **Force-velocity profile variables** |
| --- |
| Force-velocity profiles maximal values over RSA test of:   - the theorical maximal horizontal force (Max F_0_; N.kg^-1^) - the theorical maximal velocity (Max V_0_; m.s^-1^) - the maximal power developed during the sprint (Max Pmax; W.kg^-1^) |
| Changes in force-velocity profile parameters between the maximal value and the worst value of:   - the theorical maximal horizontal force (ΔF_0_; N.kg^-1^) - the theorical maximal velocity (ΔV_0_; m.s^-1^) - the maximal power developed during the sprint (ΔPmax; W.kg^-1^) |

Table 4. Independent variables related to sprint pattern of the repeated sprints.

| **Sprint pattern variables (observed at 5 m and 25 m)** |
| --- |
| Pre-fatigue peak angle   - of θS (θS_1-2_; °), calculated as the mean of θS during the first two sprints - of θF (θF_1-2_; °), calculated as the mean of θF during the first two sprints |
| In-fatigue peak angle   - of θS (θS_9-10_; °), calculated as the mean of θS during the last two sprints - of θF (θF_9-10_; °), calculated as the mean of θF during the last two sprints |
| Maximal peak angle value during the RSA test   - of θS (θS_max_; °) - of θF (θF_max_; °) |
| Changes in the peak angles with fatigue   - θS (ΔθS; °), calculated by subtracting θS_1-2_ to θS_9-10_ - θF(ΔθF; °), calculated by subtracting θF_1-2_ to θF_9-10_ |
