# Supplementary material for: Development of a preliminary multivariable model predicting hamstring strain injuries during preseason screening in soccer players: a multidisciplinary approach
Source: Ann Med. 2025 May 8;57(1):2494683. doi: 10.1080/07853890.2025.2494683 (PMC12064112; doi:10.1080/07853890.2025.2494683)
Supplement: Supplemental Material [file IANN_A_2494683_SM9750.zip › suppl_data/Supplemental online material 1.docx]

# Supplemental online material 1: Additional material and methods details

## Testing procedure

Preseason testing procedure.

The entire procedure lasted around 2h 30min per participant. First, the main investigator collected the consent forms, verified attestations of medical aptitude to practice competitive sport, and checked the inclusion criteria (i.e., over 16 years old, training at least 3 times per week, with at least 4 years’ experience playing soccer, not goalkeepers, participating voluntarily, with no psychological disorders or depression according to the Patient Health Questionnaire (PHQ-2), no current injury, and no hamstring pain). Then, anthropometric data were collected (i.e., body mass (kg), height (m)), and participants filled in a personal data form (e.g., experience in soccer, player position, status, dominant leg [defined as the kicking leg], injury history). Next, participants undertook a 20-minute warm-up routine, which included general cardiovascular activation, muscle-specific drills, and machine-specific contractions at progressive intensity. Following this warm-up, participants performed maximal voluntary isometric contractions (MVICs) of their lower limbs. After completing the knee extensor MVICs, participants alternated between 4 sessions of questionnaires completed in a dedicated space, and 3 sessions of MVICs for the knee flexors, to assess knee flexor torques. The order of the questionnaires and the muscle length position of the MVICs was randomized between participants. This strategy was adopted to prevent fatigue and minimize lassitude during tests. The testing procedure continued with a specific warm-up for sprinting, which included 5 min of running, active-dynamic stretching, running drills, and 2 accelerations. After the warm-up, a 3-min passive rest period was observed, followed by the repeated sprint ability (RSA) test. Subsequently, a reassessment of knee flexor torques was conducted.

## Questionnaires

Athletic identity.

We used the Athlete Identity Measurement Scale (AIMS),^1^ to assess the athletic identity of the participants. This 10-item questionnaire covers social identity, self-identity, negative affectivity, and exclusivity. The participants are asked to indicate the degree to which they agree with each affirmation (e.g., “Sport is the most important part of my life”) on a 7-point scale ranging from 1 (strongly disagree) to 7 (strongly agree). Higher scores indicate stronger athletic identity. The French version of the questionnaire has not been validated but was used among a French sample of competitive athletes in Caudroit et al.^2^ (Cronbach’s alpha: .86) and validated among a French-Canadian sample of para-hockey players in Laidet et al.^3^ (Cronbach’s alpha: .88). To better suit our soccer player sample, “sport” was replaced by “soccer” in all the items (e.g., “Soccer is the most important part of my life”). Cronbach’s alpha for the present study was .78.

Nutritional habits.

1. Self-rated diet***.*** We asked to the participants to self-rate their diet on a 5-point scale ranging from 1 (very unhealthy) to 5 (very healthy) using the single question: “Overall, do you think your eating habits are…”.^4^
2. Usual junk food intake. Participants were asked to report how many days in an average week they would drink soda or eat sweet snacks or confectionery, sweetened breakfast cereals, ice cream, savory snacks, and hamburgers or fast food (Cronbach’s alpha: .77).^4^ They rated each item on an 8-point scale from 0 (never) to 7 (every day). Cronbach’s alpha for the present study was .73.
3. Eating disorders. We used the French version of the Sick, Control, One, Fat, Food^5,6^ scale to detect participants susceptible to eating disorders. This is a questionnaire with 5 questions (e.g., “Do you make yourself Sick because you feel uncomfortably full?”) and dichotomic answers 0 (no) or 1 (yes). A total score ≥2 indicates a potential eating disorder (sensitivity 94.6% (83-98), specificity (94.8% (91-96)).

Inclination to play through pain.

1. Susceptibility to persist through pain. We used a 1-item measure in line with research in the injury prevention domain.^7^ Participants were asked to answer to the question: “During a soccer match, if you feel a pain that you think might lead to injury, please indicate until what pain intensity you would continue playing at the same pace” on a 7-point scale ranging from 1 (very low) to 7 (very high).
2. Subjective norms in soccer. We used a 3-item questionnaire developed following Ajzen’s^8^ recommendations, where participants indicated their degree of agreement with each affirmation (i.e., “In my sport, we don't listen to ourselves when we're tired”, “In my sport, it's not uncommon to train despite pain”, and “In my sport, sometimes we play a match with pain”) on a 7-point scale ranging from 1 (strongly disagree) to 7 (strongly agree). Cronbach’s alpha for the present study was .62.

Perceived health knowledge**.**

As there is no validated short survey about athletes’ health literacy available in French, we chose to assess the soccer players’ perception of their knowledge of the human body, health, and the value of preventive exercises with a single item question. This question was adapted from Sorg et al.’s^9^ study on “the perception of injuries by athletes and their influences on the implementation of measures to prevent injuries in athletics”, and their item 8.5 about the value of knowledge to better prevent injuries, in the “preventive strategies to prevent injuries” section. Participants were asked to indicate their degree of agreement with the affirmation “I have a good knowledge of how the human body and health work, and of the importance of injury prevention” on a 7-point scale ranging from 1 (strongly disagree) to 7 (strongly agree).

Perceived susceptibility to soccer-related injury**.**

We used the Perceived Susceptibility to Sport Injury scale (PSSI)^7,10^ adapted for soccer to measure the perceived susceptibility of suffering an injury our participants have when they are playing soccer. This 4-item questionnaire presented a single factor structure, a good invariance across gender, and a good reliability (66.85% of the variance was explained by a single factor, factor loadings ranged from .80 to .84, and Cronbach’s alpha: .83).^7,10^ Participants were asked to answer to the following questions on a 7-point scale: “What do you believe is the chance that you will get an injury while playing soccer?” from 1 (no chance of being injured) to 7 (certain to be injured); “How susceptible do you feel you will get an injury while playing soccer?” from 1 (not at all susceptible) to 7 (very susceptible); “What do you believe is the chance that you will get an injury while playing soccer?” from 1 (less than a 10% chance) to 7 (100% chance); and “What do you believe your chances are of getting an injury while playing soccer compared with other soccer players?” from 1 (a lot lower) to 7 (a lot higher). A higher total score indicated a greater perceived susceptibility to injury. Cronbach’s alpha for the present study was .82.

Achievement goals.

We used the French Achievement Goals Questionnaire for Sport and Exercise (FAGQSE)^11^ to assess the achievement goals of the participants. This 12-item questionnaire is composed of 4 subscales and presents a good to acceptable reliability for athletes: mastery-approach goals (Cronbach’s alpha: .88), performance-approach goals (Cronbach’s alpha: .92), mastery-avoidance goals (Cronbach’s alpha: .75) and performance-avoidance goals (Cronbach’s alpha: .88). The participants rated their degree of agreement with each affirmation (e.g., “My goal is to perform better than others”) on a 5-point scale ranging from 1 (strongly disagree) to 5 (strongly agree). Cronbach’s alpha values for the present study were respectively .71, .43, .84, and .76.

Sport Anxiety.

We used the French version of the Modified Sport Anxiety Scale (SAS)^12^ to evaluate the competitive anxiety of the players. This 16-item questionnaire has two factors: somatic anxiety (Cronbach’s alpha: .89) and cognitive anxiety (Cronbach’s alpha: .86). Participants were asked to indicate how much they usually experience the feelings and thoughts described in the items before or during matches (e.g., “My body feels tight”). They rated it on a 4-point scale from 1 (not at all) to 4 (very much so). A higher score indicated more competitive anxiety. Cronbach’s alpha for the present study was.74 for somatic anxiety and .84 for cognitive anxiety.

**Sleep.**

1. Sleep quality. We used the French version of the Athens Insomnia Scale (AIS-FR; composite reliability ω: 72),^13^ to assess nocturnal and diurnal manifestations of participants’ sleep disturbance. They were asked to indicate the magnitude of the sleep disturbances described in the 8 items (e.g., “awakenings during the night”) if they experienced them at least 3 times per week, and rate them in a 4-point scale from 0 (no disturbance) to 3 (severe disturbance). A higher total score indicated poorer sleep quality. Composite reliability ω for the present study was .85, Cronbach’s alpha was .80.
2. Sleep duration. We collected players’ usual sleep durations with the single-item question used in the Pittsburgh Sleep Quality Index (PSQI).^14^ To reflect the habits of the players during the season rather than during preseason and holidays we adapted the question and asked them to indicate how many hours of sleep they usually get per night during a competitive period.
3. Sleep behaviours. We used the French version of the Athlete Sleep Behaviour Questionnaire (ASBQ-FR; composite reliability ω: .67)^13^ to assess the behaviours that athletes adopted that could negatively impact their sleep. This 15-item questionnaire has 3 factors: behaviours affecting sleep, behaviours related to anxiety, and sleep disturbances. Participants were asked to indicate how frequently they engage in these specific behaviours (e.g., “I go to bed at different times each night (more than ±1 hour variation)”) in a 5-point scale from 1 (never) to 5 (always). A higher total score indicated that the athlete adopts more behaviours that can affect his/her sleep. Composite reliability ω for the present study was .61, Cronbach’s alpha was .62.

Coping strategies.

We use the French version of the Ways of Coping Checklist (WCC)^15,16^ to assess the coping strategies used by athletes to face stressful events (sport-related or not). This questionnaire has 3 distinct factors: social support (Cronbach’s alpha: .73), emotion-focused coping (Cronbach’s alpha: .72), and problem-focused coping (Cronbach’s alpha: .79). Participants were asked to remember an event which was particularly stressful for them and then indicate on a 4-point scale [1 (no), 2 (somewhat no), 3 (somewhat yes), 4 (yes)] if they had adopted the strategies described in the item (e.g., “I hoped a miracle would happen”) to face this event. Cronbach’s alpha for the present study was respectively .68 for the social support factor, .66 for emotion-focused coping factor, and .70 for the problem-focused coping factor.

Personality.

We used the 10-item version of the Big Five Inventory (BFI-10)^17^ to examine the personality traits of the participants. This questionnaire was composed of five factors: extraversion, agreeableness, conscientiousness, neuroticism, and openness to experience. Two items evaluate each factor (including 1 in reverse for each factor), which makes Cronbach’s alpha not appropriate here. Participants were asked to indicate on a 4-point scale ranging from 1 (strongly disagree) to 4 (strongly agree) how well the statements in the items describe their personality (“You see your character as someone who…” e.g., “…is reserved”).

Burnout.

We used the Athlete Burnout Questionnaire (AB0-S)^18^ to measure the athletes’ burnout. This 15-item questionnaire has 3 factors: physical exhaustion (Cronbach’s alpha: .89), reduced sense of accomplishment (Cronbach’s alpha: .79), and sport devaluation (Cronbach’s alpha: .74). Participants were asked to indicate the answer that best describes how they feel in relation to the demands of training and/or competitions (e.g., “I feel incompetent”). Answers were rated on a 5-point scale from 1 (almost never) to 5 (almost always). A higher score indicated a greater tendency to burnout. Cronbach’s alpha for the present study was respectively .70 for a reduced sense of accomplishment, .77 for sport devaluation, and .84 for physical exhaustion.

## Maximal voluntary isometric contractions

**Warm-up.**

The complete warm-up lasted for 20 min. It began with 5 min of running for the cardiovascular activation, followed by 2 sets of 6 repetitions per leg of specific hamstring exercises (i.e., “the extender” and “the diver” as described by Askling et al.,^19^ and unilateral glute bridges). Then, participants performed isometric contractions at progressive intensity levels (i.e., at 50%, 75%, 90% and one at 100% of their maximal perceived contraction) on specific ergometers before the first measured trial. These contractions also served as familiarization with the machine for the execution of the movement.

Force/torque measurements.

Fig. 1 illustrates the positions on the ergometers.

- Please insert Fig. 1 near here -

The force/torque sensors were placed 5 cm above the external lateral malleolus (on the shin or on the Achilles tendon depending on the evaluation). During each MVIC, participants were asked to “push as hard and as fast” as they could and to hold the contraction for 3 s. During knee flexor MVICs, a pre-tension of 10 N was applied by participants to prevent them from kicking the sensor. Signals were sampled at 1 kHz using an external acquisition card (PowerLab 8/35, ADInstrument, New South Wales, Australia) controlled by customized software (Labchart 8 Pro, ADInstrument, New South Wales, Australia). The higher peak force value was retained. To calculate torque for the knee flexors, force values were converted using the participants’ lever arms. These lever arms were determined as the distance between the head of the fibula and the heel contact point on the ergometer. All independent variables derived from force/torque measurements are summarized in Table 1.

- Please insert Table 1 near here -

## repeated sprints

**Warm-up.**

Sprint warm-up began by 5 min of low intensity running, followed by 2 sets of 3 active-dynamic stretching (targeting the quadriceps, hamstrings, and gluteus maximus), and 2 sets of 4 running drills (heel to bum, high knees lifts, scissor runs, B-skips), with a higher movement frequency for the second set. Next, participants performed 2 progressive accelerations until 80% and 100% of their maximal perceived speed.

**RSA test.**

Layout of the measurement equipment during the RSA test is illustrated in Fig. 2. The last cones were placed at 32 m to reduce the deceleration before the 30m photocells usually observed in RSA tests. To limit perspective bias, sagittal cameras were placed perpendicular to the running line, and frontal cameras were placed as parallel as possible to the running line. Verbal explanation of the protocol was provided before the beginning of the session. Players were asked to run with their usual soccer cleats, as fast as they could on each sprint. Standardized strong encouragements were provided throughout each run. Furthermore, players were given feedback on their time at the end of each sprint for motivation. The start was given every 30 seconds with a pre-programmed soundtrack. Thus, the player had 30 seconds to cover the 32 m sprinting and then jog back to the starting zone. A standardized three seconds of countdown were provided before each sprint start signal.

- Please insert Fig. 2 near here -

The RSA test took place directly near the strength tests to minimize the recovery time between the end of the last sprint and the post-sprint maximal voluntary isometric contractions. RSA test variables are presented in Table 2.

- Please insert Table 2 near here -

**Force-Velocity profiles.**

The horizontal velocity as a function of time (V_H_(t)) follows a mono-exponential function during maximal accelerations, directly measured by the radar in the present study (Fig. 3A). To isolate relevant data points, departure and speed plateau were manually selected and only the data in between were retained. Data were extracted and computed in the Excel file developed by Samozino et al..^21^ This file considers the inverse linear force-velocity and the parabolic power-velocity relationships to deduce the force-velocity profile of the players (Fig. 3B). The acceleration as a function of time(a_H_(t)) was calculated as the derivative of V_H_(t) over time. Horizontal external force during sprint running (F_H_(t)) was calculated from the participant’s body mass (in kg), a_H_(t), and the aerodynamic drag to overcome during sprint running F_aero_(t). To calculate F_aero_(t), the investigator provided the participants’ height (in m), air temperature (in °C), and atmospheric pressure (in mmHg). Finally, the mean net horizontal antero-posterior power output applied to the body center of mass (P_H_) as a function of time was calculated by multiplying F_H_(t) and V_H_(t). Force-Velocity variables considered in the study are presented in Table 3.

- Please insert Fig. 3 near here –

- Please insert Table 3 near here -

**Sprint patterns**.

We use the 2D markerless method OpenPose (OpenPose body_25 model, v1.7.0, https://github.com/CMU-Perceptual-Computing-Lab/openpose/releases), a markerless deep learning-based pose estimation method, to obtain body segment positions on each frame. Participants tucked their tee-shirts into their shorts to enable better estimation of the pelvic position. Then, we calculated knee and hip angles in the sagittal plane, as well as lateral pelvic tilt and shoulder tilt in the front plane using Python scripts (PyCharm 2022.1, JetBrains s.r.o., Nusle, Czech Republic) on each frame (Fig. 4).

- Please insert Fig. 4 near here -

In the sagittal plane, we retained as a variable the peak angle θS, representative of the maximal length of the hamstring during the late swing phase, calculated as Equation 3:

(Equation 3) $\theta S=\theta hip contro-\theta hip homo-\theta knee homo$

Where θknee homo and θhip homo are the angles of knee flexion and hip flexion of the lower limb doing the late swing phase (homolateral), respectively, and θhip control is the flexion angle of the contralateral hip (extension values are expressed as negatives).

In the frontal plane, we retained as a variable the peak angle θF, representative of the frontal thoraco-pelvic control during the late swing phase, calculated as the maximal angle between the shoulders and the pelvis. Values of θS and θP used as variables are presented in Table 4.

- Please insert Table 4 near here -

**References**

1. Brewer BW, van Raalte JL, Linder DE. Athletic identity: Hercules’ muscles or Achilles heel? *J Sport Psychol*. 1993;24(2):237-254.

2. Caudroit J, Stephan Y, Brewer BW, Le Scanff C. Contextual and Individual Predictors of Psychological Disengagement From Sport During a Competitive Event. *J Appl Soc Psychol*. 2010;40(8):1999-2018. doi:10.1111/J.1559-1816.2010.00648.X

3. Laidet M, Lemoyne J, Trudeau F. Understanding para hockey participation: the role of athletic identity and intentions of sport participation. *Mov Sport Sci*. 2023;121(3):1-14. doi:https://doi.org/10.1051/sm/2022032

4. Dixon HG, Scully ML, Wakefield MA, White VM, Crawford DA. The effects of television advertisements for junk food versus nutritious food on children’s food attitudes and preferences. *Soc Sci Med*. 2007;65(7):1311-1323. doi:10.1016/J.SOCSCIMED.2007.05.011

5. Garcia FD, Grigioni S, Allais E, Houy-Durand E, Thibaut F, Déchelotte P. Detection of eating disorders in patients: validity and reliability of the French version of the SCOFF questionnaire. *Clin Nutr*. 2011;30(2):178-181. doi:10.1016/J.CLNU.2010.09.007

6. Morgan JF, Reid F, Lacey JH. The SCOFF questionnaire: assessment of a new screening tool for eating disorders. *BMJ Br Med J*. 1999;319(7223):1468. doi:10.1136/BMJ.319.7223.1467

7. Chalabaev A, Radel R, Ben Mahmoud I, Massiera B, Deroche T, d’Arripe-Longueville F. Is motivation for marathon a protective factor or a risk factor of injury? *Scand J Med Sci Sports*. 2017;27(12):2040-2047. doi:10.1111/SMS.12807

8. Ajzen I. *Constructing a Theory of Planned Behavior Questionnaire*.; 2006. doi:https://people.umass.edu/aizen/pdf/tpb.measurement.pdf

9. Sorg M, Ruffault A, Martin S, et al. Étude sur la perception des blessures par les athlètes et leurs influences sur la réalisation de mesures de prévention des blessures en athlétisme. *J Traumatol du Sport*. 2020;37(4):193-200. doi:10.1016/J.JTS.2020.09.004

10. Gnacinski SL, Arvinen-Barrow M, Brewer BW, Meyer BB. Factorial validity and measurement invariance of the Perceived Susceptibility to Sport Injury scale. *Scand J Med Sci Sports*. 2017;27(7):754-761. doi:10.1111/SMS.12681

11. Riou F, Boiché J, Doron J, et al. Development and validation of the French achievement goals questionnaire for sport and exercise (FAGQSE). *Eur J Psychol Assess*. 2012;28(4):313-320. doi:10.1027/1015-5759/A000112

12. Marcel J, Paquet Y. French validation of the modified version of the SAS. *Encephale*. 2010;36(2):116-121. doi:10.1016/J.ENCEP.2009.05.006

13. Baize D, Meriaux-Scoffier S, Chrétien A, Hayotte M, Piponnier E, d’Arripe-Longueville F. Sleep Assessment in Competitive Athletes: Development and Validation of French Versions of the Athens Insomnia Scale and the Athlete Sleep Behavior Questionnaire. *Sleep Sci*. 2023;16(2):183-196. doi:10.1055/S-0043-1770803

14. Buysse DJ, Reynolds CF, Monk TH, Berman SR, Kupfer DJ. The Pittsburgh Sleep Quality Index: a new instrument for psychiatric practice and research. *Psychiatry Res*. 1989;28(2):193-213. doi:10.1016/0165-1781(89)90047-4

15. Bruchon-Schweitzer M, Cousson F, Quintard B, Nuissier J, Rascle N. French adaptation of the ways of coping checklist. *Percept Mot Skills*. 1996;85(1):104-106. doi:10.2466/PMS.1996.83.1.104

16. Cousson-Gélie F, Bruchon-Schweitzer M, Quintard B, Nuissier J, Rascle N. Analyse multidimensionnelle d’une échelle de coping : validation française de la W.C.C. (Ways of Coping checklist). *Psychol Française*. 1996;42(2):155-164.

17. Courtois R, Petot J-M, Plaisant O, et al. Validation française du Big Five Inventory à 10 items (BFI-10). *Encephale*. 2020;46(6):455-462. doi:10.1016/J.ENCEP.2020.02.006

18. Isoard-Gautheur S, Martinent G, Guillet-Descas E, Trouilloud D, Cece V, Mette A. Development and evaluation of the psychometric properties of a new measure of Athlete Burnout: The Athlete Burnout Scale. *Int J Stress Manag*. 2018;25(S1):108-123. doi:10.1037/STR0000083

19. Askling CM, Tengvar M, Thorstensson A. Acute hamstring injuries in Swedish elite football: A prospective randomised controlled clinical trial comparing two rehabilitation protocols. *Br J Sports Med*. 2013;47(15):953-959. doi:10.1136/BJSPORTS-2013-092165/-/DC1

20. Whittaker RL, Sonne MW, Potvin JR. Ratings of perceived fatigue predict fatigue induced declines in muscle strength during tasks with different distributions of effort and recovery. *J Electromyogr Kinesiol*. 2019;47:88-95. doi:10.1016/J.JELEKIN.2019.05.012

21. Samozino P, Rabita G, Dorel S, et al. A simple method for measuring power, force, velocity properties, and mechanical effectiveness in sprint running. *Scand J Med Sci Sports*. 2016;26(6):648-658. doi:10.1111/SMS.12490
