# Supplementary material for: Development of a preliminary multivariable model predicting hamstring strain injuries during preseason screening in soccer players: a multidisciplinary approach
Source: Ann Med. 2025 May 8;57(1):2494683. doi: 10.1080/07853890.2025.2494683 (PMC12064112; doi:10.1080/07853890.2025.2494683)
Supplement: Supplemental Material [file IANN_A_2494683_SM9750.zip › suppl_data/Supplemental online material 2.docx]

# HAPPY-1 Questionnaires groupe A

Consignes : Veuillez répondre sincèrement à chacune des questions posées.

Il n'existe pas de bonne ou de mauvaise réponse. Les données collectées sont anonymes et confidentielles.

Il y a 12 questions dans ce questionnaire.

## Code d'anonymat

Veuillez renseigner le code d'anonymat qui vous a été attribué.

Veuillez écrire votre réponse ici :

## Échelle de Mesure de l'identité Athlétique (AIMS-FR)

Les 10 items suivants sont à propos de vous. Veuillez sélectionner le chiffre qui reflète le mieux votre degré d'accord ou de désaccord avec chaque affirmation concernant votre pratique du football.

Vous avez 7 possibilités allant de 1 « pas du tout d'accord », à 7 « tout à fait d'accord ».

Veuillez sélectionner 10 réponses.

Choisissez la réponse appropriée pour chaque élément :

|  | Pas du tout d'accord |  |  |  |  |  | Tout à fait d'accord |
| --- | --- | --- | --- | --- | --- | --- | --- |
|  | 1 | 2 | 3 | 4 | 5 | 6 | 7 |
| 1. Je me considère comme un footballeur | 🞆 | 🞆 | 🞆 | 🞆 | 🞆 | 🞆 | 🞆 |
| 2. J'ai de nombreux objectifs liés au football | 🞆 | 🞆 | 🞆 | 🞆 | 🞆 | 🞆 | 🞆 |
| 3. La plupart de mes amis sont des footballeurs | 🞆 | 🞆 | 🞆 | 🞆 | 🞆 | 🞆 | 🞆 |
| 4. Le football occupe la part la plus importante de ma vie | 🞆 | 🞆 | 🞆 | 🞆 | 🞆 | 🞆 | 🞆 |
| 5. Je passe plus de temps à penser au football qu'à quoi que ce soit d'autre | 🞆 | 🞆 | 🞆 | 🞆 | 🞆 | 🞆 | 🞆 |
| 6. J'ai besoin de jouer au football pour me sentir bien | 🞆 | 🞆 | 🞆 | 🞆 | 🞆 | 🞆 | 🞆 |
| 7. Les autres personnes me voient principalement comme un footballeur | 🞆 | 🞆 | 🞆 | 🞆 | 🞆 | 🞆 | 🞆 |
| 8. Je me sens mal quand joue peu au football | 🞆 | 🞆 | 🞆 | 🞆 | 🞆 | 🞆 | 🞆 |
| 9. Le football est la seule chose importante dans ma vie | 🞆 | 🞆 | 🞆 | 🞆 | 🞆 | 🞆 | 🞆 |
| 10. Je serais vraiment déprimé si je me blessais et que je ne pouvais pas jouer un match de football | 🞆 | 🞆 | 🞆 | 🞆 | 🞆 | 🞆 | 🞆 |

## Habitudes alimentaires

En général, vous pensez que vos habitudes alimentaires sont :

Veuillez sélectionner une réponse ci-dessous

Veuillez sélectionner une seule des propositions suivantes :

🞆 Très malsaines

🞆 Assez malsaines

🞆 Ni saines, ni malsaines

🞆 Plutôt saines

🞆 Très saines

En moyenne, combien de **jours par semaine** consommez-vous :

Veuillez sélectionner 6 réponses

Choisissez la réponse appropriée pour chaque élément :

|  | 0 | 1 | 2 | 3 | 4 | 5 | 6 | Tous les jours |
| --- | --- | --- | --- | --- | --- | --- | --- | --- |
| Du soda | 🞆 | 🞆 | 🞆 | 🞆 | 🞆 | 🞆 | 🞆 | 🞆 |
| Des snacks sucrés ou des confiseries | 🞆 | 🞆 | 🞆 | 🞆 | 🞆 | 🞆 | 🞆 | 🞆 |
| Des céréales sucrées | 🞆 | 🞆 | 🞆 | 🞆 | 🞆 | 🞆 | 🞆 | 🞆 |
| De la glace | 🞆 | 🞆 | 🞆 | 🞆 | 🞆 | 🞆 | 🞆 | 🞆 |
| Des chips ou biscuits apéro | 🞆 | 🞆 | 🞆 | 🞆 | 🞆 | 🞆 | 🞆 | 🞆 |
| Des hamburgers ou fast-food | 🞆 | 🞆 | 🞆 | 🞆 | 🞆 | 🞆 | 🞆 | 🞆 |

Répondez aux questions suivantes

Veuillez sélectionner 5 réponses

Choisissez la réponse appropriée pour chaque élément :

|  | Oui | Non |
| --- | --- | --- |
| Vous faites-vous vomir lorsque vous avez une sensation de "trop plein" ? | 🞆 | 🞆 |
| Êtes-vous inquiet d'avoir perdu le contrô1e des quantités que vous mangez ? | 🞆 | 🞆 |
| Avez-vous récemment perdu plus de 6kg en moins de 3 mois ? | 🞆 | 🞆 |
| Vous trouvez-vous gros alors même que les autres disent que vous êtes trop mince ? | 🞆 | 🞆 |
| Diriez-vous que la nourriture domine votre vie ? | 🞆 | 🞆 |

## Normes de récupération

Pendant un match de football :

Si tu ressens une douleur qui pourrait conduire à une blessure, indique jusqu'à quelle intensité de douleur tu pourrais continuer à jouer au même rythme.

Veuillez choisir une réponse

Choisissez la réponse appropriée pour chaque élément :

| Très faible  1 | 2 | 3 | 4 | 5 | 6 | Très élevée  7 |
| --- | --- | --- | --- | --- | --- | --- |
| 🞆 | 🞆 | 🞆 | 🞆 | 🞆 | 🞆 | 🞆 |

Normes subjectives au football :

Veuillez sélectionner le chiffre qui reflète le mieux votre degré d'accord ou de désaccord avec chacune des affirmations suivantes.

Veuillez sélectionner 3 réponses

Choisissez la réponse appropriée pour chaque élément :

|  | Pas du tout d’accord  1 | 2 | 3 | 4 | 5 | 6 | Tout à fait d’accord  7 |
| --- | --- | --- | --- | --- | --- | --- | --- |
| Dans mon sport, on ne s'écoute pas quand on est fatigué | 🞆 | 🞆 | 🞆 | 🞆 | 🞆 | 🞆 | 🞆 |
| Dans mon sport, il n'est pas rare de s'entrainer malgré des douleurs | 🞆 | 🞆 | 🞆 | 🞆 | 🞆 | 🞆 | 🞆 |
| Dans mon sport, il arrive que l'on fasse un match avec une douleur | 🞆 | 🞆 | 🞆 | 🞆 | 🞆 | 🞆 | 🞆 |

## Connaissances

Veuillez sélectionner le chiffre qui reflète le mieux votre degré d'accord ou de désaccord avec l'affirmation suivante.

Veuillez choisir une réponse

Choisissez la réponse appropriée pour chaque élément :

|  | Pas du tout d’accord  1 | 2 | 3 | 4 | 5 | 6 | Tout à fait d’accord  7 |
| --- | --- | --- | --- | --- | --- | --- | --- |
| J'ai de bonnes connaissances sur le fonctionnement du corps humain et de la santé, ainsi que sur l'intérêt de la prévention des blessures | 🞆 | 🞆 | 🞆 | 🞆 | 🞆 | 🞆 | 🞆 |

## Susceptibilité perçue à la blessure liée à la pratique du football

Veuillez sé1ectionner le chiffre correspondant à votre réponse pour chacune des questions suivantes

Selon toi, quelle est la chance que tu te blesses en jouant au football ?

Choisissez la réponse appropriée pour chaque élément :

|  | Aucune chance de me blesser  1 | 2 | 3 | 4 | 5 | 6 | Certain(e) de me blesser  7 |
| --- | --- | --- | --- | --- | --- | --- | --- |
|  | 🞆 | 🞆 | 🞆 | 🞆 | 🞆 | 🞆 | 🞆 |

Dans quelle mesure es-tu susceptible de te blesser en jouant au football ?

Choisissez la réponse appropriée pour chaque élément :

|  | Pas du tout susceptible  1 | 2 | 3 | 4 | 5 | 6 | Très susceptible  7 |
| --- | --- | --- | --- | --- | --- | --- | --- |
|  | 🞆 | 🞆 | 🞆 | 🞆 | 🞆 | 🞆 | 🞆 |

Selon toi, quelle est ta probabilité de te blesser en jouant au football ?

Choisissez la réponse appropriée pour chaque élément :

|  | Moins de 10% de chance  1 | 2 | 3 | 4 | 5 | 6 | 100% de chance  7 |
| --- | --- | --- | --- | --- | --- | --- | --- |
|  | 🞆 | 🞆 | 🞆 | 🞆 | 🞆 | 🞆 | 🞆 |

Selon toi, quelles sont les chances que tu te blesses en jouant au football par rapport aux autres joueurs ?

Choisissez la réponse appropriée pour chaque élément :

|  | Beaucoup moins  1 | 2 | 3 | 4 | 5 | 6 | Beaucoup plus  7 |
| --- | --- | --- | --- | --- | --- | --- | --- |
|  | 🞆 | 🞆 | 🞆 | 🞆 | 🞆 | 🞆 | 🞆 |

# HAPPy-1 Questionnaires groupe B

Consignes : Veuillez répondre sincèrement à chacune des questions posées.

II n'existe pas de bonne ou de mauvaise réponse. Les données collectées sont anonymes et confidentielles.

II y a 5 questions dans ce questionnaire.

## Code d'anonymat

Veuillez renseigner le code d'anonymat qui vous a été attribué.

Veuillez écrire votre réponse ici :

## Questionnaire Français des Buts d'Accomplissement pour le Sport et l'Exercice Physique (QFBASEP)

« Lorsque tu pratiques ton sport, quels sont tes objectifs ou tes buts ? »

Veuillez répondre sincèrement à cette question en sélectionnant la valeur qui représente le mieux votre degré d'accord avec chacune des affirmations suivantes.

Choisissez la réponse appropriée pour chaque élément :

|  | 1 : Pas du tout d'accord | 2 | 3 | 4 | 5 : Tout à fait d'accord |
| --- | --- | --- | --- | --- | --- |
| 1. Mon but est de progresser autant que possible. | 🞆 | 🞆 | 🞆 | 🞆 | 🞆 |
| 2. Je cherche à ne pas faire les choses à moitié. | 🞆 | 🞆 | 🞆 | 🞆 | 🞆 |
| 3. Je cherche à éviter d'être moins bon(ne) que les autres. | 🞆 | 🞆 | 🞆 | 🞆 | 🞆 |
| 4. Je cherche à réaliser le mieux possible ce que je dois faire. | 🞆 | 🞆 | 🞆 | 🞆 | 🞆 |
| 5. Mon but est d'être meilleur(e) que les autres. | 🞆 | 🞆 | 🞆 | 🞆 | 🞆 |
| 6. Je cherche à éviter d'être en-dessous des autres. | 🞆 | 🞆 | 🞆 | 🞆 | 🞆 |
| 7. Mon but est de m'améliorer le plus possible. | 🞆 | 🞆 | 🞆 | 🞆 | 🞆 |
| 8. Mon but est de surpasser les autres. | 🞆 | 🞆 | 🞆 | 🞆 | 🞆 |
| 9. Je cherche à éviter de mal faire les choses. | 🞆 | 🞆 | 🞆 | 🞆 | 🞆 |
| 10. Mon objectif est d'éviter de faire moins bien que les autres. | 🞆 | 🞆 | 🞆 | 🞆 | 🞆 |
| 11. Je cherche à être au-dessus des autres. | 🞆 | 🞆 | 🞆 | 🞆 | 🞆 |
| 12. Mon but est d'éviter de faire des erreurs. | 🞆 | 🞆 | 🞆 | 🞆 | 🞆 |

## Échelle d'anxiété sportive (« Sport Anxiety Scale »)

Un certain nombre de déclarations que les sportifs utilisent pour décrire leurs pensées et leurs sensations avant et pendant la compétition sont listées ci-dessous. Lisez chaque déclaration et sélectionnez ensuite l'affirmation à droite de la déclaration qui correspond le mieux à la façon dont vous vous sentez généralement en compétition.

Il n'y a donc pas de bonne ou de mauvaise réponse. Ne passez pas trop de temps sur chaque déclaration.

Choisissez la réponse appropriée pour chaque élément :

|  | Pas du tout | Un peu | Modérément | Beaucoup |
| --- | --- | --- | --- | --- |
| 1. Je me sens nerveux(se) | 🞆 | 🞆 | 🞆 | 🞆 |
| 2. Je doute de moi | 🞆 | 🞆 | 🞆 | 🞆 |
| 3. Mon corps est contracté | 🞆 | 🞆 | 🞆 | 🞆 |
| 4. Je suis préoccupé(e) à l'idée de ne pas faire aussi bien que j'en suis capable en compétition | 🞆 | 🞆 | 🞆 | 🞆 |
| 5. Je ressens des tensions dans mon estomac | 🞆 | 🞆 | 🞆 | 🞆 |
| 6. Penser que je pourrais réaliser une performance médiocre perturbe ma concentration pendant la compétition | 🞆 | 🞆 | 🞆 | 🞆 |
| 7. Je suis préoccupé(e) à l'idée de craquer sous la pression | 🞆 | 🞆 | 🞆 | 🞆 |
| 8. Mon cœur s’emballe | 🞆 | 🞆 | 🞆 | 🞆 |
| 9. Je sens mon estomac se retourner | 🞆 | 🞆 | 🞆 | 🞆 |
| 10. Je suis préoccupé(e) à l'idée de réaliser une performance médiocre | 🞆 | 🞆 | 🞆 | 🞆 |
| 11. Je me retrouve parfois à trembler avant ou pendant une compétition | 🞆 | 🞆 | 🞆 | 🞆 |
| **12.** Je suis inquiet(e) par rapport à l'atteinte de mon but | 🞆 | 🞆 | 🞆 | 🞆 |
| 13. Mon corps est crispé | 🞆 | 🞆 | 🞆 | 🞆 |
| 14. Je suis préoccupé(e) à l'idée que les autres puissent être déçus de ma performance | 🞆 | 🞆 | 🞆 | 🞆 |
| 15. Mon estomac est dérangé avant ou pendant une compétition | 🞆 | 🞆 | 🞆 | 🞆 |
| 16. Mon cœur bat fort avant une compétition | 🞆 | 🞆 | 🞆 | 🞆 |

Échelle d'insomnie d'Athènes (AIS-FR)

Cette échelle est destinée à évaluer votre perception de vos perturbations de sommeil.

Pour chacun des items ci-dessous, veuillez indiquer votre estimation de l'importance ces perturbations.

Seules les perturbations s'étant manifestées au moins trois fois par semaine au cours du dernier mois doivent être indiquées.

NB : nous n'évaluons pas ici la fréquence de chaque perturbation mais son importance.

ex : Question 3 "Réveil matinal plus tôt que l'heure fixée"

- si je me réveille habituellement à l'heure normale -> je ne réponds "aucune perturbation",
- si je me réveille habituellement un peu plus tôt que prévu -> je réponds "perturbations mineurs",
- si je me réveille habituellement bien plus tôt que prévu -> je réponds "perturbations importantes"
- si je me réveille habituellement très en avance ou ne dors pas -> je réponds "perturbations sévères"

Importance de mes perturbations de sommeil s'étant produites au moins trois fois par semaine au cours du dernier mois

Choisissez la réponse appropriée pour chaque élément :

|  | **Aucune perturbation** | **Perturbations mineures** | **Perturbations importantes** | **Perturbations sévères** |
| --- | --- | --- | --- | --- |
| 1. DIFFICULTES D'ENDORMISSEMENT (difficultés à m'endormir après avoir éteint la lumière) | 🞆 | 🞆 | 🞆 | 🞆 |
| 2. REVEILS PENDANT LA NUIT | 🞆 | 🞆 | 🞆 | 🞆 |
| 3. REVEIL MATINAL PLUS TOT QUE L'HEURE FIXEE | 🞆 | 🞆 | 🞆 | 🞆 |
| 4. DUREE TOTALE DE SOMMEIL INSUFFISANTE | 🞆 | 🞆 | 🞆 | 🞆 |
| 5. SOMMEIL DE MAUVAISE QUALITE (i.e. sensation d'avoir mal dormi, peu importe le temps que j'ai dormi) | 🞆 | 🞆 | 🞆 | 🞆 |
| 6. SENSATION DE BIEN-ETRE DIMINUEE PENDANT LA JOURNEE (sans lien avec une contrariété ou un tracas) | 🞆 | 🞆 | 🞆 | 🞆 |
| 7. ACTIVITE (PHYSIQUE ET MENTALE) DIMINUEE  PENDANT LA JOURNEE (sans raison évidente) | 🞆 | 🞆 | 🞆 | 🞆 |
| 8. SOMNOLENCE / ENVIE DE DORMIR DURANT LA JOURNEE | 🞆 | 🞆 | 🞆 | 🞆 |

## Durée du sommeil

En période compétitive, en moyenne, combien d'heures dormez-vous par nuit ?

Veuillez écrire votre réponse ici :

# HAPPy-1 Questionnaires groupe C

Consignes : Veuillez répondre sincèrement à chacune des questions posées.

II n'existe pas de bonne ou de mauvaise réponse. Les données collectées sont anonymes et confidentielles.

II y a 3 questions dans ce questionnaire.

## Code d'anonymat

Veuillez renseigner le code d'anonymat qui vous a été attribué.

Veuillez écrire votre réponse ici :

## Ways of Coping Checklist (WCC)

Repensez à un évènement récent qui vous a particulièrement stressé, bouleversé, troublé (avec ou sans lien avec votre pratique sportive). Pour chacune des stratégies proposées ci-dessous, veuillez indiquer si vous les avez utilisées pour faire face à cet évènement.

Choisissez la réponse appropriée pour chaque élément :

|  | Non | Plutôt non | Plutôt oui | Oui |
| --- | --- | --- | --- | --- |
| 1. J'ai établi un plan d'action et je l'ai suivi. | 🞆 | 🞆 | 🞆 | 🞆 |
| 2. J'ai souhaité que la situation disparaisse ou finisse. | 🞆 | 🞆 | 🞆 | 🞆 |
| 3. J'ai parlé à quelqu'un de ce que je ressentais. | 🞆 | 🞆 | 🞆 | 🞆 |
| 4. Je me suis battu pour ce que je voulais. | 🞆 | 🞆 | 🞆 | 🞆 |
| 5. J'ai souhaité pouvoir changer ce qui est arrivé. | 🞆 | 🞆 | 🞆 | 🞆 |
| 6. J'ai sollicité l'aide d'un professionnel et j'ai fait ce qu'on m'a conseillé. | 🞆 | 🞆 | 🞆 | 🞆 |
| 7. J'ai changé positivement. | 🞆 | 🞆 | 🞆 | 🞆 |
| 8. Je me suis senti(e) mal de ne pas pouvoir éviter le problème. | 🞆 | 🞆 | 🞆 | 🞆 |
| 9. J'ai demandé des conseils à une personne digne de respect et je les ai suivis. | 🞆 | 🞆 | 🞆 | 🞆 |
| 10. J'ai pris les choses unes par unes. | 🞆 | 🞆 | 🞆 | 🞆 |
| 11. J'ai espéré qu'un miracle se produirait. | 🞆 | 🞆 | 🞆 | 🞆 |
| 12. J'ai discuté avec quelqu'un pour en savoir plus au sujet de la situation. | 🞆 | 🞆 | 🞆 | 🞆 |
| 13. Je me suis concentré(e) sur un aspect positif qui pourrait apparaitre après. | 🞆 | 🞆 | 🞆 | 🞆 |
| 14. Je me suis culpabilisé(e). | 🞆 | 🞆 | 🞆 | 🞆 |
| 15. J'ai contenu (gardé pour moi) mes émotions. | 🞆 | 🞆 | 🞆 | 🞆 |
| 16. Je suis sorti(e) plus fort(e) de la situation. | 🞆 | 🞆 | 🞆 | 🞆 |
| 17. J'ai pensé à des choses irréelles ou fantastiques pour me sentir mieux. | 🞆 | 🞆 | 🞆 | 🞆 |
| 18. J'ai parlé avec quelqu'un qui pouvait agir concrètement au sujet du problème. | 🞆 | 🞆 | 🞆 | 🞆 |
| 19. J'ai changé des choses pour que tout puisse bien finir. | 🞆 | 🞆 | 🞆 | 🞆 |
| 20. J'ai essayé de tout oublier. | 🞆 | 🞆 | 🞆 | 🞆 |
| 21. J'ai essayé de ne pas m'isoler. | 🞆 | 🞆 | 🞆 | 🞆 |
| 22. J'ai essayé de ne pas agir de manière précipitée ou de suivre la première idée. | 🞆 | 🞆 | 🞆 | 🞆 |
| 23. J'ai souhaité pouvoir changer d'attitude. | 🞆 | 🞆 | 🞆 | 🞆 |
| 24. J'ai accepté la sympathie et la compréhension de quelqu'un. | 🞆 | 🞆 | 🞆 | 🞆 |
| 25. J'ai trouvé une ou deux solutions au problème. | 🞆 | 🞆 | 🞆 | 🞆 |
| 26. Je me suis critiqué(e) ou sermonné(e). | 🞆 | 🞆 | 🞆 | 🞆 |
| 27. Je savais ce qu'il fallait faire, aussi j'ai redoublé d'efforts et j'ai fait tout mon possible pour y arriver. | 🞆 | 🞆 | 🞆 | 🞆 |

## Big Five Inventory à 10 items (BFl-10)

Instructions :

Vous allez trouver un certain nombre de qualificatifs qui peuvent ou non s'appliquer à vous. Par exemple, acceptez-vous d'être quelqu'un qui aime passer du temps avec les autres ? Sélectionnez l'affirmation qui vous correspond par rapport à chacune des situations suivantes.

Je me vois comme quelqu'un qui...

Choisissez la réponse appropriée pour chaque élément :

|  | **Désapprouve fortement** | **Désapprouve un peu** | **N'approuve ni ne désapprouve** | **Approuve un peu** | **Approuve fortement** |
| --- | --- | --- | --- | --- | --- |
| **_est réservé** | 🞆 | 🞆 | 🞆 | 🞆 | 🞆 |
| _**a tendance à critiquer les autres** | 🞆 | 🞆 | 🞆 | 🞆 | 🞆 |
| **_travaille consciencieusement** | 🞆 | 🞆 | 🞆 | 🞆 | 🞆 |
| **_est « relaxe », détendu, gère bien le stress** | 🞆 | 🞆 | 🞆 | 🞆 | 🞆 |
| _**a une grande imagination** | 🞆 | 🞆 | 🞆 | 🞆 | 🞆 |
| _**est sociable, extraverti** | 🞆 | 🞆 | 🞆 | 🞆 | 🞆 |
| _**fait généralement confiance aux autres** | 🞆 | 🞆 | 🞆 | 🞆 | 🞆 |
| _**a tendance à être paresseux** | 🞆 | 🞆 | 🞆 | 🞆 | 🞆 |
| **_est facilement anxieux** | 🞆 | 🞆 | 🞆 | 🞆 | 🞆 |
| _**est peu intéressé par tout ce qui est artistique** | 🞆 | 🞆 | 🞆 | 🞆 | 🞆 |

# HAPPy-1 Questionnaires groupe D

Consignes : Veuillez répondre sincèrement à chacune des questions posées.

II n'existe pas de bonne ou de mauvaise réponse. Les données collectées sont anonymes et confidentielles.

II y a 3 questions dans ce questionnaire.

## Code d'anonymat

Veuillez renseigner le code d'anonymat qui vous a été attribué.

Veuillez écrire votre réponse ici :

## Le questionnaire de comportement de sommeil des athlètes (ASBQ-FR)

Cette échelle est destinée à évaluer vos comportements de sommeil.

Pour chaque item, veuillez sélectionner la réponse représentant le mieux vos comportements de sommeil ces derniers temps (au cours du dernier mois)...

Choisissez la réponse appropriée pour chaque élément :

|  | Jamais | Rarement | Parfois | Souvent | Toujours |
| --- | --- | --- | --- | --- | --- |
| Je fais des siestes l'après-midi de deux heures ou plus | 🞆 | 🞆 | 🞆 | 🞆 | 🞆 |
| J'utilise des stimulants lorsque je m'entraine/participe à une compétition (ex : la caféine) | 🞆 | 🞆 | 🞆 | 🞆 | 🞆 |
| Je consomme de l'alcool ou des boissons stimulantes (ex : cola, boissons énergisantes, the...) dans les 4 heures avant d'aller au lit | 🞆 | 🞆 | 🞆 | 🞆 | 🞆 |
| Je vais au lit a des heures différentes chaque soir (variation de plus de ± 1 heure) | 🞆 | 🞆 | 🞆 | 🞆 | 🞆 |
| Je vais au lit en ayant soif | 🞆 | 🞆 | 🞆 | 🞆 | 🞆 |
| Je vais au lit avec des douleurs musculaires | 🞆 | 🞆 | 🞆 | 🞆 | 🞆 |
| J'utilise des appareils e1ectroniques émettant de la lumière dans l'heure précédant l'endormissement (ex : ordinateur portable, smartphone, télévision, jeux vidéo) | 🞆 | 🞆 | 🞆 | 🞆 | 🞆 |
| Je pense (ex : réflexions, planification) et m'inquiète au sujet de ma performance sportive quand je suis au lit | 🞆 | 🞆 | 🞆 | 🞆 | 🞆 |
| Je pense (ex : réflexions, planifications) et m'inquiète à propos de problèmes non liés à mon sport quand je suis au lit | 🞆 | 🞆 | 🞆 | 🞆 | 🞆 |
| J'utilise des somnifères/comprimés pour m'aider à dormir | 🞆 | 🞆 | 🞆 | 🞆 | 🞆 |
| Je me réveille pour aller aux toilettes plus d'une fois par nuit | 🞆 | 🞆 | 🞆 | 🞆 | 🞆 |
| Je me réveille et/ou je réveille mon/ma partenaire avec mes ronflements | 🞆 | 🞆 | 🞆 | 🞆 | 🞆 |
| Je me réveille et/ou je réveille mon/ma partenaire à cause de secousses musculaires involontaires dans mes membres | 🞆 | 🞆 | 🞆 | 🞆 | 🞆 |
| Je me 1eve a des heures différentes chaque matin (variation de plus de ± 1 heure) | 🞆 | 🞆 | 🞆 | 🞆 | 🞆 |
| Chez moi, je dors dans un environnement qui n'est pas idéal (ex : trop lumineux, trop bruyant, lit/oreiller inconfortable, trop chaud/froid) | 🞆 | 🞆 | 🞆 | 🞆 | 🞆 |

## Athlete Burnout Scale (ABO-S)

Nous voulons savoir ce que vous pensez de votre implication dans votre sport. II est important que vous répondiez à toutes les questions.

Sélectionnez une affirmation par phrase. II n'y a pas de bonnes ou de mauvaises réponses. Ne passez pas trop de temps sur une des affirmations, mais choisissez la réponse qui décrit le mieux ce que vous pensez par rapport aux exigences des entrainements et/ou des compétitions.

Choisissez la réponse appropriée pour chaque élément :

|  | Presque jamais | Rarement | Quelques fois | Fréquemment | Souvent |
| --- | --- | --- | --- | --- | --- |
| 1. Je suis incapable de réussir une bonne performance | 🞆 | 🞆 | 🞆 | 🞆 | 🞆 |
| 2. Je me sens lassé(e) | 🞆 | 🞆 | 🞆 | 🞆 | 🞆 |
| 3. Je me sens en réussite | 🞆 | 🞆 | 🞆 | 🞆 | 🞆 |
| 4. J’ai des sentiments négatifs envers mon sport | 🞆 | 🞆 | 🞆 | 🞆 | 🞆 |
| 5. Il me semble que quoique je fasse, je suis en échec | 🞆 | 🞆 | 🞆 | 🞆 | 🞆 |
| 6. Je suis crevé(e) physiquement | 🞆 | 🞆 | 🞆 | 🞆 | 🞆 |
| 7. Je me sens à bout de nerf | 🞆 | 🞆 | 🞆 | 🞆 | 🞆 |
| 8. Je me sens faible physiquement | 🞆 | 🞆 | 🞆 | 🞆 | 🞆 |
| 9. Je n’arrive pas à grand-chose | 🞆 | 🞆 | 🞆 | 🞆 | 🞆 |
| 10. Je me sens vidé(e) physiquement | 🞆 | 🞆 | 🞆 | 🞆 | 🞆 |
| 11. Je ressens du dégoût envers mon sport | 🞆 | 🞆 | 🞆 | 🞆 | 🞆 |
| 12. J’ai l’impression que mes batteries sont à plat | 🞆 | 🞆 | 🞆 | 🞆 | 🞆 |
| 13. J’ai l’impression d’être incompétent(e) | 🞆 | 🞆 | 🞆 | 🞆 | 🞆 |
| 14. Je me sens agacé(e) | 🞆 | 🞆 | 🞆 | 🞆 | 🞆 |
| 15. Je manque d’énergie | 🞆 | 🞆 | 🞆 | 🞆 | 🞆 |
