# Supplementary material for: Development of a preliminary multivariable model predicting hamstring strain injuries during preseason screening in soccer players: a multidisciplinary approach
Source: Ann Med. 2025 May 8;57(1):2494683. doi: 10.1080/07853890.2025.2494683 (PMC12064112; doi:10.1080/07853890.2025.2494683)
Supplement: Supplemental Material [file IANN_A_2494683_SM9750.zip › suppl_data/Supplemental online material 3.docx]

# Supplemental online material 3: Group descriptive statistics

| Independent variables | Group | | | | | | Mean difference between groups | |
| --- | --- | --- | --- | --- | --- | --- | --- | --- |
|  | Uninjured | | | Injured | | | p-value (T-test or Mann-Whitney U-test) | |
| Sex | 16♀ (18%) / 75♂ (82%) | | | 6♀ (21%) / 23♂ (79%) | | |  | |
| HSI history | 14 (17%) | | | 10 (34%) | | |  | |
| Age (years) | 17.8±2.6 | | | 19.7±5.1 | | | .106^M^ | |
| **Questionnaires** | Results | Imputations | | Results | Imputations | |  | |
| Athletic identity (AIMS-FR_/7) | 5.8±.9 | / | | 5.8±.8 | / | | .906 | |
| Self-rated diet (/5) | 3.7±.8 | / | | 3.6±.6 | / | | .415 | |
| Usual junk food intake (days per week) | 1.5±1.0 | / | | 1.5±1.1 | / | | .985 | |
| Eating disorders (SCOFF_/5) | .40±.71 | / | | .28±.59 | / | | .415 | |
| Susceptibility to persist through pain (/7) | 4.4±1.4 | / | | 4.3±1.1 | / | | .917 | |
| Subjective norms (/21) | 13.9±4.2 | / | | 15.4±3.3 | / | | **.085** | |
| Perceived health knowledge (/7) | 4.6±1.4 | / | | 4.2±1.4 | / | | .265 | |
| Perceived vulnerability (/28) | 12.5±3.6 | / | | 14.3±4.8 | / | | **.072** | |
| Achievements goals (FAGQSE) | | (1) | |  | (2) | |  | |
| FAGQSE_mastery-approach goals (/15) | 14.8±.8 |  | | 14.6±.9 |  | | .678^M^ | |
| FAGQSE_performance-approach goals (/15) | 13.8±2.0 |  | | 12.8±3.4 |  | | .154 | |
| FAGQSE_mastery-avoidance goals (/15) | 13.9±1.7 |  | | 14.0±1.6 |  | | .855 | |
| FAGQSE_performance-avoidance goals (/15) | 13.0±2.6 |  | | 12.2±3.9 |  | | .342 | |
| Sport Anxiety (SAS_/64) | 24.7±6.2 | (1) | | 26.0±7.9 | (2) | | .359 | |
| Sleep quality (AIS-FR_/24) | 4.9±3.7 | (1) | | 5.2±4.1 | (2) | | .759 | |
| Sleep duration (h) | 8.0±.9 | (1) | | 8.0±.9 | (2) | | .727 | |
| Sleep behaviours (ASBQ-FR_/75) | 29.3±5.2 | (1) | | 30.9±6.1 | / | | .166 | |
| Coping strategies (WCC) | | (1) | |  | / | |  | |
| WCC_social support (/32) | 19.8±5.2 |  | | 20.3±4.9 |  | | .628 | |
| WCC_emotion-focused (/36) | 22.5±4.8 |  | | 21.7±5.8 |  | | .502 | |
| WCC_problem-focused (/40) | 32.3±4.0 |  | | 30.6±6.0 |  | | .156 | |
| Personality (BFI-10) | | (1) | |  | / | |  | |
| BFI-10_Extraversion (/8) | 6.1±2.1 |  | | 6.3±1.9 |  | | .641 | |
| BFI-10_Agreeableness (/8) | 7.3±1.6 |  | | 7.5±1.8 |  | | .585 | |
| BFI-10_Conscientiousness (/8) | 7.8±1.7 |  | | 7.7±2.1 |  | | .896 | |
| BFI-10_Neuroticism (/8) | 4.5±2.0 |  | | 4.5±2.3 |  | | .899 | |
| BFI-10_Openness (/8) | 6.9±1.8 |  | | 6.5±1.8 |  | | .280 | |
| Burnout (ABO-S_/75) | 28.0±7.0 | (1) | | 27.6±8.7 | / | | .793 | |
| ABO-S_physical exhaustion (/25) | 10.5±3.4 |  | | 10.5±4.2 |  | | .953 | |
| ABO-S_reduced sense of accomplishment (/25) | 9.3±2.5 |  | | 9.2±3.1 |  | | .857 | |
| ABO-S_sport devaluation (/25) | 8.2±2.6 |  | | 7.9±3.5 |  | | .708 | |
| **Maximal voluntary isometric contractions** | | | | | | | | |
|  | Dom | Non-dom | | Dom | Non-dom | | Dom | Non-dom |
| Tmax_KE_ (N.m) | 271.2±66.1 | 278.2±72.0 | | 261.8±63.5 | 271.6±77.0 | | .503 | .670 |
| Imputations | / | / | | / | / | |  |  |
| Tmax_KF_ at SL (N.m) | 118.8±30.4 | 106.6±28.4 | | 132.9±39.5 | 108.2±41.5 | | .045 | .845 |
| Imputations | / | / | | / | / | |  |  |
| Tmax_KF_ at IL (N.m) | 137.6±33.9 | 122.6±34.0 | | 151.7±44.3 | 123.6±33.3 | | .124 | .895 |
| Imputations | / | / | | / | / | |  |  |
| Tmax_KF_ at LL (N.m) | 143.4±33.2 | 128.8±34.7 | | 157.1±40.8 | 130.1±39.7 | | .070 | .775 |
| Imputations | (1) | / | | (1) | / | |  |  |
| Imb_KE_ (% Dom) | 12.0±9.4 | | | 15.8±14.8 | | | .239^M^ | |
| Imb_KF_ at SL (% Dom) | 26.3±16.4 | | | 30.6±11.4 | | | .127 | |
| Imb_KF_ at IL (% Dom) | 24.6±16.6 | | | 27.3±20.1 | | | .471 | |
| Imb_KF_ at LL (% Dom) | 20.8±15.3 | | | 25.5±14.0 | | | .147 | |
| **Repeated sprints** | Results | | Imputations | Results | | Imputations |  | |
| Best sprint (s) | 4.4±.2 | | / | 4.3±.2 | | / | **.036** | |
| RSA_Index_ (%) | 6.9±3.8 | | (1) | 8.8±6.4 | | / | **.055^M^** | |
| ΔPerceived fatigue (/10) | 4.7±1.7 | | (1) | 4.7±2.0 | | / | .854 | |
| Max F_0_ (N.kg^-1^) | 9.9±1.0 | | (17) | 9.7±.7 | | / | .218 | |
| Max V_0_ (m.s^-1^) | 8.8±.7 | | (17) | 9.1±.8 | | / | **.035** | |
| Max Pmax (W.kg^-1^) | 1308.6±297.4 | | (17) | 1394.0±278.1 | | / | .174 | |
| ΔF_0_ (%) | 30.4±10.3 | | (17) | 31.4±12.5 | | / | .667 | |
| ΔV_0_ (%) | 16.5±7.1 | | (17) | 18.7±9.2 | | / | .190 | |
| ΔPmax (%) | 31.9±9.2 | | (17) | 33.7±11.4 | | / | .376 | |
|  | Dom | Non-dom | | Dom | Non-dom | | Dom | Non-dom |
| Tmax_KF_ in Post1 (N.m) | 134.8±31.6 | 116.9±33.2 | | 136.6±36.1 | 115.0±34.2 | | .799 | .795 |
| Imputations | (3) | (3) | | (1) | (1) | |  |  |
| Tmax_KF_ in Post2 (N.m) | 131.3±33.7 | 115.4±31.9 | | 139.8±32.6 | 113.3±34.2 | | .237 | .766 |
| Imputations | (4) | (1) | | / | (1) | |  |  |
| ΔTmax_KF_ in Post1 (%) | -4.8±12.3 | -8.3±15.7 | | -11.7±11.2 | -11.4±12.4 | | **.008** | .324 |
| ΔTmax_KF_ in Post2 (%) | -7.6±14.3 | -9.2±16.2 | | -8.6±11.4 | -12.6±12.4 | | .731 | .303 |
|  | 5m | 25m | | 5m | 25m | | 5m | 25m |
| θS_max_ (°) | 79.0±14.7 | 56.0±16.8 | | 78.2±12.7 | 57.1±17.3 | | .821 | .776 |
| Imputations | (8) | (7) | | (4) | (1) | |  |  |
| θS_1-2_ (°) | 69.2±9.6 | 41.0±10.3 | | 67.7±7.4 | 39.7±9.2 | | .484 | .549 |
| Imputations | (9) | (8) | | (5) | (1) | |  |  |
| θS_9-10_ (°) | 67.8±11.6 | 36.5±11.5 | | 68.3±10.5 | 38.8±12.6 | | .849 | .399 |
| Imputations | (12) | (12) | | (6) | (5) | |  |  |
| ΔθS (°) | -1.6±9.5 | -4.8±12.6 | | .1±8.1 | -1.7±13.8 | | .396 | .271 |
| Imputations | (12) | (13) | | (7) | (5) | |  |  |
| θF_max_ (°) | 24.8±4.3 | 24.4±3.9 | | 24.3±3.9 | 24.4±4.0 | | .555 | .990 |
| Imputations | (7) | (7) | | (1) | (2) | |  |  |
| θF_1-2_ (°) | 17.8±4.2 | 17.3±3.8 | | 18.9±4.2 | 17.6±3.5 | | .235 | .738 |
| Imputations | (7) | (7) | | (1) | (2) | |  |  |
| θF_9-10_ (°) | 18.8±4.3 | 17.8±3.6 | | 18.6±3.8 | 18.3±3.4 | | .878 | .557 |
| Imputations | (8) | (8) | | (1) | (2) | |  |  |
| ΔθF (°) | .9±4.7 | .5±4.0 | | -.3±3.6 | .7±3.7 | | .151 | .830 |
| Imputations | (8) | (8) | | (1) | (2) | |  |  |

^M^= p-value with U-test of Mann-Whitney; bold=p≤.10.

**Abbreviations:** Dom=dominant lower limb; Non-dom=non-dominant lower limb.

**Units:** °=degrees; %=percentage; %Dom=percentage of the dominant lower limb measure; kg=kilograms; m=meters; N=newtons; s=seconds; W=watts.

**Questionnaires:** ABO-S= Athlete Burnout Questionnaire; AIMS=Athlete Identity Measurement; AIS-FR= French version of the Athens Insomnia Scale; ASBQ-FR=French version of the Athlete Sleep Behaviour Questionnaire; BFI-10=10-items version of the Big Five Inventory; FAGQSE=French Achievement Goals Questionnaire for Sport and Exercise; Perceived vulnerability=perceived susceptibility to soccer-related injury; SAS=Modified Sport Anxiety Scale; SCOFF=Scale Sick, Control, One, Fat, Food; Sleep duration=usual sleep duration in competitive period; Subjective norms=subjective norms in soccer regarding pain and fatigue; WCC=Ways of Coping Checklist.

**Maximal voluntary isometric contractions:** KE=knee extensors; KF=knee flexors; IL=intermediate hamstring muscle length; imb=imbalance between the dominant and the non-dominant lower limb; LL= long hamstring muscle length; Tmax=maximal peak torque; SL=short hamstring muscle length.

**Repeated sprints:** 1-2=mean of the first two sprint values; 9-10=mean of the last two sprint values; ΔPerceived fatigue=absolute change in the perceived fatigue between pre- and post-sprints values; Δθ= changes in angles with fatigue; ΔTmax_KE_=knee extensors performance fatigability; ΔTmax_KF_= knee flexors performance fatigability; F_0_= theorical maximal horizontal force; max=maximal value; Pmax=maximal mechanical power; Post1=first post-sprint torque measurement; Post2=second post-sprint torque measurement; RSA_Index_=repeated sprint ability index; V_0_= theorical maximal velocity; θF= angle in the frontal plane between the shoulders and the pelvis (a higher value representing less trunk control); θS =composite value of the peak angles in the sagittal plane of homolateral knee flexion; contralateral hip flexion; and homolateral hip flexion angles (a higher value representing more tension on the hamstrings).

**Note.** AIS-FR: bad sleep quality if total score >6; SCOFF: presence of eating disorders if total score≥2.
