# Supplementary figures and images for: Development of a preliminary multivariable model predicting hamstring strain injuries during preseason screening in soccer players: a multidisciplinary approach
Source: Ann Med. 2025 May 8;57(1):2494683. doi: 10.1080/07853890.2025.2494683 (PMC12064112; doi:10.1080/07853890.2025.2494683)

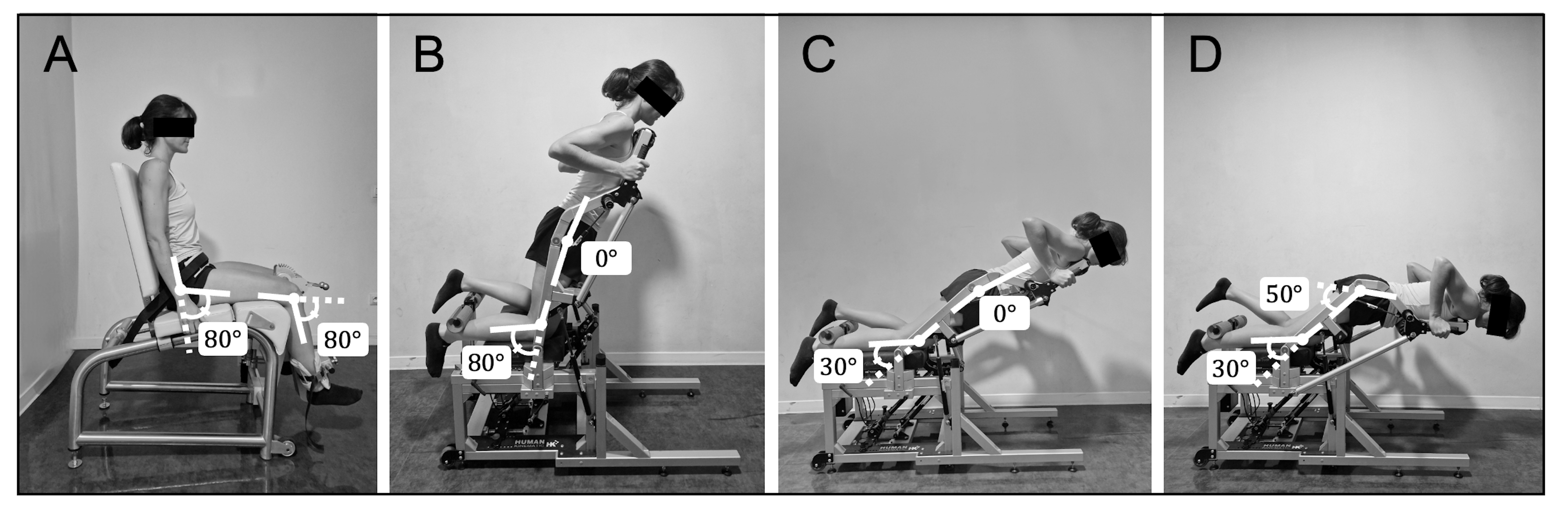

Supplement: Supplemental Material [file IANN_A_2494683_SM9750.zip › suppl_data/Supp1 Figure 1.tiff]

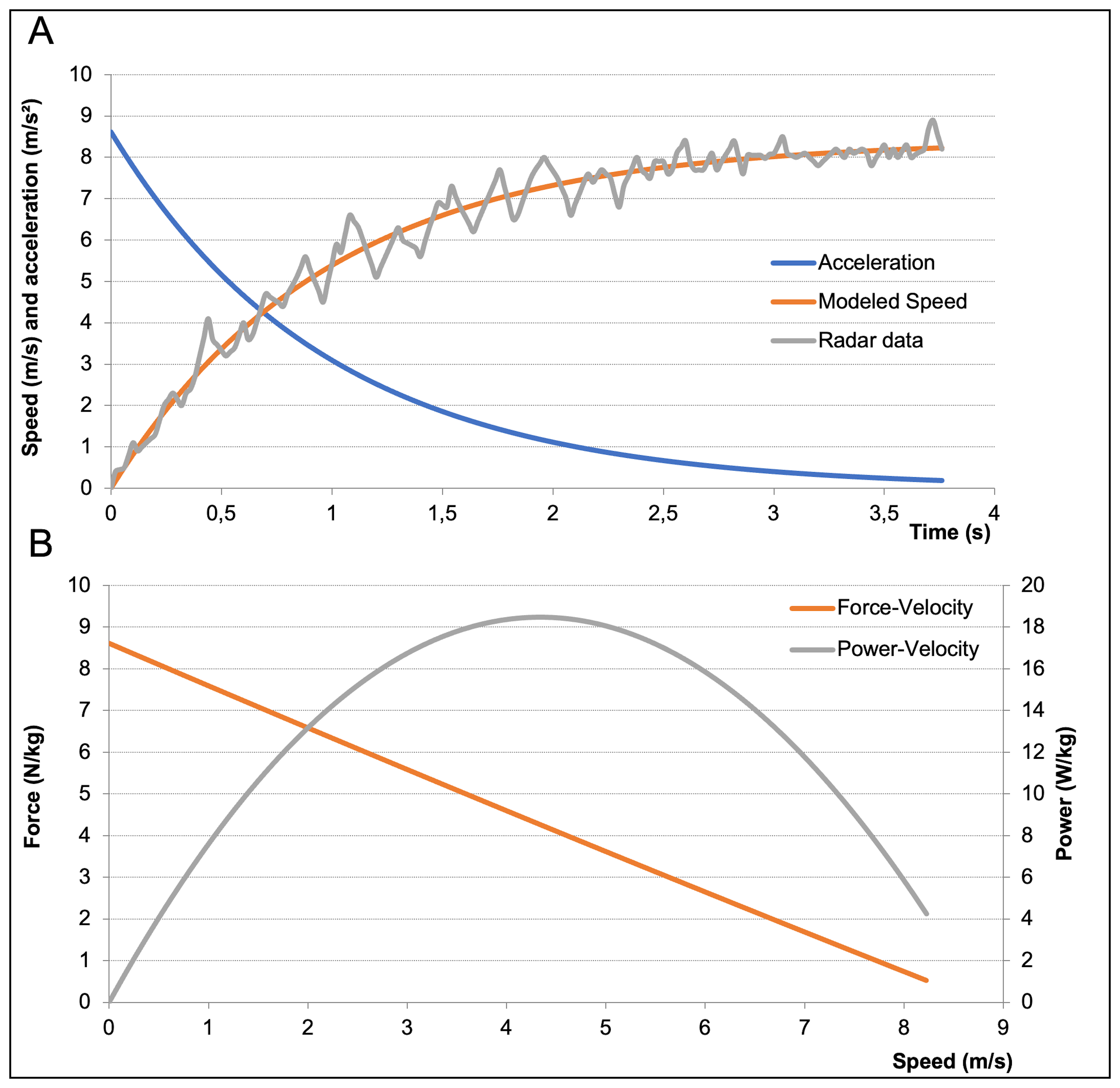

Supplement: Supplemental Material [file IANN_A_2494683_SM9750.zip › suppl_data/Supp1 Figure 3.tiff]
